# Modeling Metal(loid)s Transport in Arid Mountain Headwater Andean Basin: A WASP-Based Approach

**DOI:** 10.3390/w17131905

**Published:** 2025-06-26

**Authors:** Daniela Castillo, Ricardo Oyarzún, Pablo Pastén, Christopher D. Knightes, Denisse Duhalde, José Luis Arumí, Jorge Núñez, José Antonio Díaz

**Affiliations:** 1 Doctorate Program in Energy, Water and Environment, Universidad de La Serena, La Serena 1700000, Chile; 2 Mining Engineering Department, Universidad de La Serena, La Serena 1700000, Chile; 3 Water Research Center for Agriculture and Mining (Centro de Recursos Hídricos para la Agricultura y la Minería, CRHIAM), ANID FONDAP Center, Universidad de Concepción, Concepción 4070411, Chile; 4 Center for Advanced Studies in Arid Zones (Centro de Estudios Avanzados en Zonas Áridas, CEAZA), La Serena 1700000, Chile; 5 Department of Hydraulic and Environmental Engineering, Pontificia Universidad Católica de Chile, Macul 7810000, Chile; 6 Sustainable Urban Development Center (Centro de Desarrollo Urbano Sustentable, CEDEUS), Providencia 7500000, Chile; 7 Atlantic Coastal Environmental Sciences Division, Center for Environmental Measurement & Modeling, Office of Research and Development, United States Environmental Protection Agency, Narragansett, RI 02882, USA; 8 Doctorate Program in Water Resources and Energy for Agriculture, Universidad de Concepción, Chillán 3812120, Chile; 9 Department of Water Resources, Universidad de Concepción, Chillán 3812120, Chile

**Keywords:** modeling, metal(loid)s, WASP, water quality, acid rock drainage (ARD), local sensitivity

## Abstract

The occurrence of toxic metal(loid)s in surface freshwater is a global concern due to its impacts on human and ecosystem health. Conceptual and quantitative metal(loid) models are needed to assess the impact of metal(loid)s in watersheds affected by acid rock drainage. Few case studies have focused on arid and semiarid headwaters, with scarce hydrological and hydrochemical information. This work reports the use of WASP8 (US EPA) to model Al, Fe, As, Cu, and SO_4_^2−^ concentrations in the Upper Elqui River watershed in north–central Chile. Calibrated model performance for total concentrations was “good” (25.9, RRMSE; 0.7, R^2^-d) to “very good” (0.8–0.9, R^2^-d). The dissolved concentrations ranged between “acceptable” (56.3, RRMSE), “good” (28.6, RRMSE; 0.7 d), and “very good” (0.9, R^2^-d). While the model validation achieved mainly “very good” (0.8–0.9, R^2^-d) predictions for total concentrations, the predicted dissolved concentrations were less accurate for all indicators. Sensitivity analysis showed that the partition coefficient is a sensitive constant for estimating dissolved concentrations, and that integrating sorption and sediment interaction reduces the model error. This work highlights the need for detailed and site-specific information on the reactive and hydrodynamic properties of suspended solids, which directly impact the partition coefficient, sedimentation, and resuspension velocity calibration.

## 1. Introduction

The mining process can affect water quality by releasing and mobilizing elements and compounds that can be easily transported downstream, potentially affecting ecosystems and human health [1–4]. Mining increases the exposure of sulfide-rich minerals to weathering agents such as water, atmospheric oxygen, and microorganisms. This exposure causes the minerals to oxidize and dissolve, producing acidic waters with high concentrations of metals and sulfates, a process known as acid rock drainage (ARD) [5–7].

Moreover, climate change and its effect on temperature regimes and hydrological processes represents an additional factor that can affect water composition and quality [8,9]. For instance, Zarroca et al. [10] highlight that rising temperatures and severe droughts in the last decade in the mountainous basin of Noguera de Vallferrera (Central Pyrenees, Spain) have intensified natural ARD. This condition has been identified in other mountain basins, demonstrating a greater vulnerability to rising temperatures and their adverse effects on water quality, such as in the southern Rocky Mountains (Colorado, USA) [11,12], where the melting of frozen ground is probably one of the main drivers of the sulfide weathering acceleration (increasing metal loads) [13].

Similar cases have also been reported in the Himalayas [14], the European Alps [15–18], and the Yukon Territory (Canada) [17,19,20]. Additionally, Shrestha et al. [1] identified increased mining waste erosion processes associated with higher precipitation and stream discharge, resulting in an increment of metal(loid)s loads in surface water bodies. As a result of the increased frequency and severity of droughts in the mining-affected upper Nant Cwmnewydion catchment (Wales, United Kingdom), Byrne et al. [9] found attenuation of Pb and Zn in the water column during exceptionally low flows. However, the sediments are enriched on (and become a dangerous source of) toxic substances that could eventually be released during floods.

Based on the aforementioned works, it can be identified that the transport and fate of contaminants, associated with mining activity in river systems, are governed by complex interactions between several hydrogeochemical processes [7,21–23]. In this context, transport and fate models are versatile and useful tools for assessing the quality of water bodies [24]. In particular, estimating the fate and transport of elements can be addressed under two approaches [2]. The first one is based on complex chemical balance models, involving sorption processes, analysis of the speciation of the elements, calculation of saturation indexes, and batch tests at the laboratory level, among other elements to consider. The second approach addresses the problem from a simplified point of view, where the effect of the physicochemical conditions of the river system is grouped into a single factor, sorption, which is defined by the partition coefficient (K_d_) [2,25]. This coefficient describes the phase distribution of a metal(loid) concerning the surface of suspended solid particles or sediments present in the river system [2,26–29].

Previous studies in the Elqui basin, the area of interest of the current work, presented mass balances and estimates using the USGS aqueous geochemical software PHREEQC (which stands for PH Redox Equilibrium, version 2) for concentrations of Cu, Fe, and As in a 110 km system [30]. Likewise, Cubillos [31] performed a preliminary modeling using the EPA’s Water Quality Analysis Simulation (WASP) program to analyze As, Fe, and Cu concentrations over a distance of approximately 78 km. However, it presented important limitations by not considering the sorption process and overestimated calculations for the dispersion coefficient. In another study, Rossi et al. [32] evaluated a conservative mixing model for Cu, Fe, As, and SO_4_^2−^ at the upper and middle part of the Elqui basin (an 80 km realm). These three studies represent general trends in constituent behavior. However, the estimates relied on monthly averages of total concentrations and flow rates that did not always overlap, leading to potential uncertainty in the concentration estimates. Therefore, seeking greater representativeness in the input data, hydraulic parameters, and processes that participate in the non-conservative behavior of metal(loid)s is necessary.

With this in mind, the primary motivation of this work was to implement a descriptive modeling scheme that would enhance the results achieved from the oversimplified approaches previously used in the area while avoiding the use of a highly demanding input data approach. Hydrodynamic and hydrochemical data are generally scarce in arid headwater mountain basins, such as those of north–central Chile [24,33]. In this context, the study focuses on the application of the WASP8 model, specifically using the advanced toxicant module, to simulate the behavior of the metal(loid)s Al, Fe, As, and Cu, estimating K_d_ and evaluating how well the model predicts total and dissolved concentrations when compared with observations in the upper watershed of the Elqui River (hereinafter UWER). The research aims to provide a tool that represents an adequate trade-off between performance and data/input requirements, which is particularly relevant in the incipient status of the development of environmental quality standards for inland water bodies in countries such as Chile and elsewhere, especially in areas with current or abandoned mining activities, in a context of climate change.

## 2. Materials and Methods

### 2.1. Study Area

**Figure 1.**
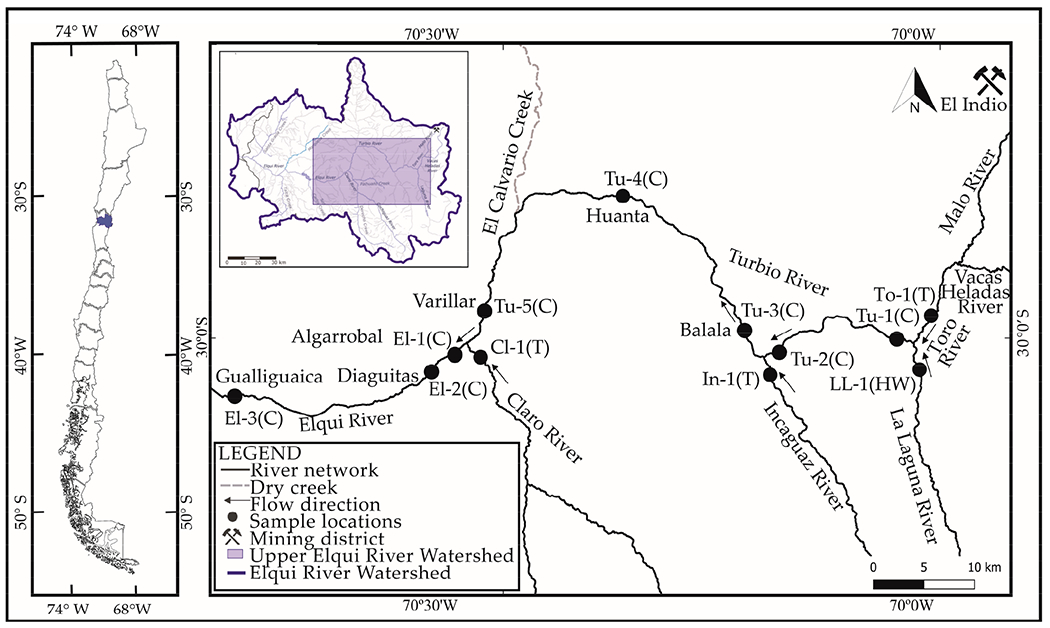
Study area and sampling locations in the UWER. To: Toro River location; LL: La Laguna River location; Tu: Turbio River locations; Cl: Claro River location; El: Elqui River locations. HW: headwater, initial location of the modeling network; T: tributaries; C: control locations.

Figure 1 shows the study area, which corresponds to the UWER, Coquimbo Region, north–central Chile. Complementary pictures of the different sections are included in the Supplementary Materials (Figure S1). The mountainous basin has river courses with moderate to high slopes, up to 7% [34].

The climate is semi-arid, as a result of the presence of the high-pressure system of the Pacific subtropical anticyclone and orographic conditions. Additionally, it is influenced by phenomena such as the Niño—Southern Oscillation (ENSO) and the Pacific Decadal Oscillation (PDO). For example, the ENSO alternates periods of intense droughts (La Niña), with a reduction of up to 60% of rainfall, and on the other hand, with intense rains (El Niño) with an increase of up to two to three times the expected average rainfall [35,36].

The hydrological regime is mostly snow-dominated. Thus, most of the precipitation occurs in winter (May–August), reaching an average of about 200–300 mm/year [37]. During the spring–summer months (October–January), snowmelt contributes to flow peaks [38]. However, since 2010, the effects of the “central Chile megadrought” [39,40] have influenced the water deficit in the basins of the northern zone [41–43], recording flows corresponding to 31% of the historical average (1991–2024) in the last five years [44], maintaining the study area’s status as a water scarcity zone since 2014 [45].

The geology of the Elqui river basin comprises several rock units, from Paleozoic to Quaternary. Specifically, the high-altitude domain includes a series of volcanic sequences of the Cenozoic age. Two of these are of particular relevance: (a) the Doña Ana Formation (Upper Oligocene–Lower Miocene), with rhyolites, rhyolitic tuffs, andesites and basaltic andesites, and (b) the Infiernillo Unit (Lower Miocene). Additionally, several (i.e., more than thirty) large hydrothermal alteration zones are present within a N–S belt of ~200 × 20 km, many of which exhibit advanced argillic alteration mineral assemblages, with kaolinite, alunite, and silica jaspers [46,47]. Thus, the area, particularly that of the Toro River, has conditions favorable for the natural development of ARD [46]. In addition, ARD generation has been enhanced by anthropogenic factors, such as the past development (1981–2002) of the El Indio-Tambo mining district located in the Andes Mountain range, located in the Malo River sub-basin [48,49]. The ore body is mainly characterized by massive enargite–pyrite–alunite and alunite–quartz veins with native sulfur in the upper levels of the deposit (copper phase) and Au–quartz ore (gold phase) [46]. This acid drainage condition is reflected in the system by the contribution of the Malo River, which flows into the Toro River. Previous assessments have shown, for the latter pH in the range of 4 to 5, high total concentrations of As (0.1–0.4 mg/L), Cu (7–10 mg/L), SO_4_^2−^ of about 1000 mg/L, and electrical conductivity of about 1 to 2 mS/cm. [50,51]. Thus, the water system is enriched in metal(loid)s in the water column and sediments. On the other hand, tributaries from the southeast (i.e., La Laguna, Incaguaz, and Claro) present mostly alkaline pH (7–8) [50,51] conditions with a lower metal(loid) content, thus diluting their presence throughout the basin [51–53]. These mixed conditions between tributaries with different water qualities provide the study area with particular interest as it becomes a “natural laboratory” to study the behavior of metal(loid)s. In fact, studies have indicated that confluences that mix tributaries affected by acid drainage with others of better quality play a significant role in the attenuation of metal(loid)-enriched particles from the water column [51–53].

The current main economic activities in the area are agriculture and livestock, which are developed mainly in the Elqui middle sub-basin (lower part of the Turbio River) [54]. Additionally, the Elqui basin is the primary source of the water resources that supply the urban and rural population of the provinces of La Serena, Coquimbo, and Vicuña (ca. 570,000 inhabitants [55,56]).

### 2.2. Field Data

#### 2.2.1. Synoptic Campaigns 2018 and 2019

To obtain information on the total and dissolved contents of Al, Fe, As, Cu and SO_4_^2−^ for the water column and the total contents for sediments, two synoptic sampling campaigns were carried out in September 2018 and January 2019. Each campaign lasted three days and coincided with the end of winter and mid-summer, respectively, representing moderate and base flow conditions. Along with water and sediment sampling, river hydromorphological data (width, depth, velocity, cross-sectional area, and flow rate) were recorded. Additionally, pH, electrical conductivity, temperature and turbidity were recorded with a Hanna Instruments HI 9829 probe (accuracies of ±0.02 pH, ±1 μS/cm, ±0.15 °C, and ±0.3 FNU, respectively). A total of 12 sampling locations were distributed along the UWER (Figure 1).

Water samples were collected manually and stored in 1 L polyethylene bottles with HNO_3_ as a preservative for total concentration of metal(loid)s and no preservative for major cations and anions. To obtain the dissolved concentrations, the samples were previously filtered using a 0.45 μm cellulose acetate membrane (47 mm diameter) and stored in 1 L polyethylene bottles with HNO_3_ as a preservative. The samples were handled and transported according to the Chilean Standard NCh 411/6 of 98 [57], which is in accordance with the International Standard ISO 5667-6:2014 [58]. Subsequently, they were refrigerated at 4 °C in the Universidad de La Serena Environmental Laboratory. Finally, the samples were sent to the Geoquímica analytical services laboratory (Coquimbo, Chile) to get the results for total and dissolved concentrations of the constituents following the “Standard Methods for the examination of water and wastewater” [59]. The metal(loid) analyses were performed using atomic absorption, and SO_4_^2−^ was analyzed using gravimetry. Sediment samples were collected from the first 10 cm of the streambed under the water column and collected in plastic containers to be preprocessed in the laboratory of the Universidad de La Serena in order to collect the fine part by sieving them to 63 μm (for more details, the reader is referred to Oyarzún et al. [46]). Subsequently, the sediments were analyzed by ICP-OES by Activation Labs (Coquimbo, Chile) using Agilent 700 Series radial equipment.

#### 2.2.2. Tracer Injection Campaign

To estimate the hydraulic parameters of the study area, such as the longitudinal dispersion coefficient (D_L_), flow rate (Q), watercourse cross-sectional area (A), transient storage zone cross-sectional area (AS), and storage zone exchange coefficient (α), a series of tracer tests were carried out in August 2019. The tracer used was common salt (NaCl), injected at six locations, and the field data were analyzed using One-Dimensional Transport with Inflow and Storage software (OTIS 1998 release, Runkel [60]). The reader is referred to Castillo et al. [61] for more details.

#### 2.2.3. Confluence Sampling Campaign 2020

In January 2020, a specific and delimited analysis was carried out at the confluences between the Toro-La Laguna and Turbio-Incaguaz rivers. Similarly to the synoptic campaigns, hydraulic variables, physicochemical parameters, and water column constituent concentrations were determined. This campaign also estimated total suspended solids (TSS) concentrations at the sampling locations, which is necessary input data for the model.

For TSS estimation, acid-free samples were filtered using a 0.45 μm cellulose acetate membrane (47 mm diameter, Merck Millipore, Burlington, MA, USA). Subsequently, the filters were dried at 100 °C for one hour and weighed on an analytical balance. After establishing the linear relationship between TSS and turbidity [62] using data from the 2020 campaign (see Supplementary Materials, Table S1), it was possible to estimate TSS for the 2018 (winter) and 2019 (mid-summer) campaigns based on their respective turbidity data.

### 2.3. Modeling Approach

#### 2.3.1. WASP Model

WASP is a dynamic mass balance program for modeling water quality, fate, and the transport of environmental contaminants, including solid particles and nanoparticles, in surface water bodies and bottom sediment layers. Governing concentration equations are based on the advection–dispersion–reaction equation between segments that form the modeling network. The model provides different spatial resolutions (one, two, and three dimensions) as the user requires. WASP was developed in 1970 by HydroScience Inc., and subsequently, in 1981, it became available for public use (US EPA, United States Environmental Protection Agency), and currently presents its latest version, WASP8 [63–65].

While the major use of WASP for constituents such as nitrogen, phosphorus, and dissolved oxygen, is well described in the literature [63–77], fewer cases exist for its use in modeling metals. Applications of the WASP model in mountain watersheds impacted by mining were initially developed by B.S. Caruso in response to assessment and restoration needs in the upper Tenmile Creek watershed, Montana, USA [27,78–81]. Later, this module was applied to transport metal(loid)s in different study areas [4,8,24,75,82–85].

For hydrodynamic modeling of advective–dispersive transport, WASP solves the general mass balance equation for constituents entering and leaving a given discrete control volume or segment using the one-dimensional finite difference method (see Equation (1)).


(1) $$\frac{\partial }{\partial \text{t}}(\text{AC})=\frac{\partial }{\partial \text{x}}\left({\text{-U}}_{\text{x}}{\text{AC+D}}_{\text{L}}\text{A}\frac{\partial \text{C}}{\partial \text{x}}\right)+\text{A}\left({\text{S}}_{\text{L}}{\text{+S}}_{\text{B}}\right){\text{+AS}}_{\text{K}}$$

where

C, metal(loid) concentration, mg/L

T, time (day)

D_L_, longitudinal dispersion coefficient, m^2^/s

U_x_, longitudinal advective velocity, m/s

A, cross-sectional area, m^2^

S_L_, direct or diffuse loading rate, g/m^3^ per day

S_B_, edge loading rate (includes upstream, downstream, benthic, and atmospheric) g/m^3^ per day

S_K_, total kinetic transformation rate (includes sources and sinks), g/m^3^ per day.

The model requires the input for the partition ratio (R_p_, see Equation (2)) as an initial condition. The R_p_ is a dimensionless parameter that varies from segment to segment. It internally calculates the dissolved fraction for each water and sediment column segment, indirectly reflecting the influence of pH and partition coefficient (K_d_).


(2) $$R_{p}=\frac{C_{p}\left(\frac{mg}{L}\right)}{C_{d}\left(\frac{mg}{L}\right)}$$

where C_p_ is the concentration of a specific metal(loid) in the particulate phase in the water column and C_d_ is the concentration of a particular metal(loid) in the dissolved phase in the water column.

The surface flow transport mode used is called stream routing (SR). This uses the flow rate and hydraulic coefficients, which vary according to the morphology of the cross-sectional area: width (W), depth (D), and segment length (L) of the channel entered in each segment. On the other hand, the volume (V) and velocity (U) are adjusted to this flow.

#### 2.3.2. Modeling Framework

The transport of the water quality constituents (Al, Fe, As, Cu, and SO_4_^2−^) of the present study was simulated using a constant flow with the advanced toxicant module for one dimension. This model consists of the water column systems (total and dissolved concentrations) and solids (total suspended solids and total sediment concentrations). It is important to note that the advance toxicant module does not consider the direct influence of pH and ionic strength. Therefore, this process is captured by sorption instead of simulating precipitation, simplifying the way in which the precipitated metals are adsorbed directly onto the particles. This may cause deficiencies in the modeling of mineral Fe and Al phases [2]. However, this simplification has been described as adequate in systems with pH ranges between 5 and 8. As the acid tributary (Rio Toro) was considered a boundary condition, the water modeled network ranges around pH 8. Thus, it is also feasible to consider global pH-related parameters as representative of the entire modeled network, such as the partition coefficient (K_d_) (which describes the sorption process) [80].

Regarding the flow rates, the total concentrations of each metal(loid) and (TSS) contributed by the headwaters and tributaries are used as boundary conditions. In particular, the flow rates were estimated and simplified to the sum of the headwater flow rate and the consecutive contributions from the downstream tributaries, in the current case, those of the Toro, Incaguaz, and Claro rivers. Other possible flow rates or calculations of individual flow rates based on the control locations of the synoptic sampling were not considered. On the other hand, the present study does not include measurements of groundwater inputs. However, as surface runoff is the main water source in the study area [86], it is possible to neglect this input in the simplified conceptual model.

#### 2.3.3. Conceptual Model

The model aims to replicate the measured concentrations by advective–dispersive transport, geochemical sorption, and hydrodynamic processes through sedimentation velocity (V_s_) and resuspension (V_r_), respectively. However, it does not consider sediment transport or sediment load modeling. The conceptual model of the present study considers a system affected by acid drainage, which is addressed with the simplified approach in which the sorption process groups all of the effects of the physicochemical conditions that affect the behavior of the metal(loid)s, represented by the global partition coefficient (K_d_) [2,25].

The sorption process follows the assumption of local chemical equilibrium between solutes and sorbents. As this implies that chemical reactions of most inorganic solutes occur rapidly and instantaneously, compared with advection–dispersion transport processes, this simplification can be assumed for metal(loid) fate and transport modeling in rivers and streams [64,87–89]. On the other hand, each constituent behaves differently given its particular nature, hydrochemical conditions, interactions, and affinities; therefore, the time to reach equilibrium or steady state varies among them. Consequently, to ensure this condition, the results were considered for one month of modeling (see Supplementary Materials, Figure S2 and Table S2).

Among the metal(loid)s modeled in this study, As and Cu were considered due to their high mobility and concentrations in the basin. Al and Fe form oxides and oxyhydroxides that act as a surface with a high affinity with As and Cu, favoring sorption and precipitation processes under specific physicochemical conditions. Furthermore, As and Cu are particularly relevant due to their potential toxicity characteristics, being generally found above the limits in water quality regulations and global average concentrations [46], causing low water quality indices in the upper Elqui river basin [90]. Additionally, SO_4_^2−^ was considered a tracer type as it behaves conservatively in the upper basin [30,32], thus identifying the effect of tributary dilution on the main network.

#### 2.3.4. Segmentation of the Study Area

**Figure 2.**
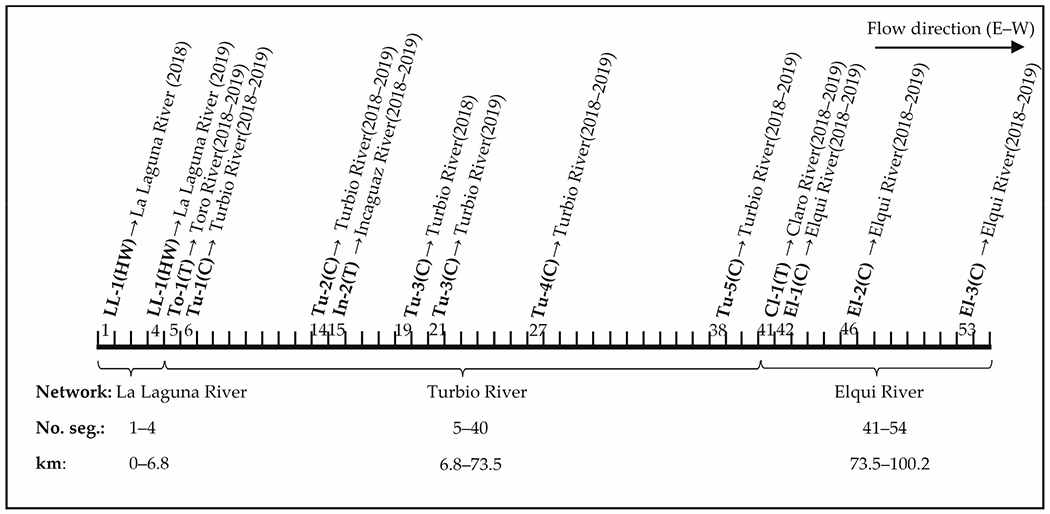
Schematic diagram for UWER with segmentation in the WASP8 model. HW: headwater, initial location of the modeling network; T: tributaries; C: control locations for model performance evaluation. No. seg.: segment number.

The main modeling network was represented by 54 water column segments totaling 100.2 km (Figure 2). For each segment, the respective morphological and hydraulic characteristics were considered (see Supplementary Materials, Tables S3–S7). The network extends from the headwaters (HW) at La Laguna River (HW: LL-1), and downstream, five locations were located in the Turbio River (C: Tu-1, Tu-2, Tu-3, Tu-4, Tu-5) and three in the Elqui River (C: El-1, El-2, and El-3). Only the segments representing these nine control locations include the water column and the surface benthic zone as sediments. Additionally, three tributaries were considered (T: Toro To-1, Incaguaz In-1, Claro Cl-1 rivers). The representative segments of the confluences are those in which the tributaries (identified with the letter T) are located, as shown in Figure 2.

The La Laguna River was considered as the headwater as its pH difference is smaller than that of the rest of the Turbio–Elqui network. Therefore, the Toro, Incaguaz, and Claro rivers are the tributaries, considered as the boundary conditions of the modeled system (Figure 2).

#### 2.3.5. Parameters and Constants

The parameters and constants related to the solids transport process were selected based on the particle diameter value of 12.7 μm, silt type classification. This diameter value corresponds to the median value obtained from the values observed in a January 2020 campaign described by Díaz et al. [51] (La Laguna River, 16 μm; Toro River, 7.7 and 7.3 μm; Turbio River, 12.7 μm; and Incaguaz River, 19.5 μm). In this same context, the range for calibration of the sedimentation velocity (V_s_) varied between 0–50 m/d (depending on the particle size), and the final calibration value was found to be close to the lower limit, with a value of 0.5 m/d for silt. The range of flow velocities for the study area was 0.7–0.9 m/s, a condition for the transport and erosion of particles. Therefore, the resuspension velocity (V_r_) in the literature for this range was 10^−4^ m/d.

Similarly, for the K_d_, the calibration began with reference values for each metal(loid) from the bibliographic review (see Supplementary Materials, Table S8), selecting those that reported the best model performance. Given several factors influencing the value of this constant, the calibrated values are consistent with those reported in the literature, ranging within the expected order of magnitude for each constituent. It is important to highlight that one of the limitations of this study is the lack of representative data for each station. As a result, we could not analyze the seasonal variability of metal(loid) behavior or estimate parameters (such as D_L_, K_d_, V_r_, and V_s_) that accurately represent the study area across different seasons or periods of varying flow. Additionally, to improve K_d_ estimation accuracy, future studies should include direct measurements of TSS throughout the watershed, along with other factors known to affect K_d_, such as particle diameter.

In particular, for the longitudinal dispersion coefficient (D_L_), as indicated by Castillo et al. [61], it was identified that the empirical formula proposed by Fischer et al. [51] is the most representative of the study area. Consequently, the D_L_ was calculated directly from the field data without calibration.

All parameters and constants used in the model, along with their variation ranges and the selected or calibrated values, are detailed in the Supplementary Materials (Tables S9 and S10).

#### 2.3.6. Calibration and Validation

Data from the September 2018 campaign were used for model calibration because they presented the most unfavorable scenario, i.e., higher total concentrations of Al, Fe, As, Cu and SO_4_^2−^, and lower flow rates, compared with the data from the 2019 campaign. Therefore, the data from the 2019 campaign were considered for validation using the calibrated parameters, i.e., partition coefficient (K_d_), sedimentation velocity (V_s_), and resuspension velocity (V_r_).

The calibration process evaluated the performance through statistical indicators and graphical analysis of the modeled total/dissolved concentrations of constituents, flow rates, and velocities. The model was applied repeatedly to select the best performance, varying the parameters to calibrate K_d_, V_s_, and V_r_.

#### 2.4. Model Performance Assessment

A combination of quantitative (statistical errors) and qualitative (graphical analysis) indicators were considered to assess the performance of the model. Concentrations of constituents, flows, and velocities from the eight control locations (Tu-1, Tu-2, Tu-3, Tu-4, Tu-5, El-1, El-2, and El-3) were considered in this analysis. The model performance indicators evaluated included the relative root mean square error (RRMSE), the coefficient of determination (R^2^) and the index of agreement (d), all of which are described in the Supplementary Materials (Section S9). In order to facilitate the interpretation, the indicator results are classified in Table 1.

The best-performing model was identified by selecting the model that achieved the highest category indicator, with at least two indicators in the same category. The middle-performance classification was considered if the model resulted in three different categories. The final ranking of each constituent was based on the same comparison of the three calculated indicators. Finally, to complement the interpretation of the model performance and identify the trends and spatial variation throughout the study area, graphs such as longitudinal profile and “1:1” scatterplot between observed and modeled data were also considered.

### 2.5. Sensitivity Analysis Description

In addition to the modeling, a sensitivity analysis was performed to evaluate the response of the model to disturbances of the calibrated parameter values (K_d_, V_s_, and V_r_), the boundary conditions (flow rate (Q)) and total suspended solids (TSS). This methodology follows the hypothesis described in Lindenschmidt et al. [84], which states that sensitivity (s, Equation (3)) increases with higher model complexity, while model error (ε, Equations (4) and (5)) decreases with increasing complexity.


(3) $$s=\frac{1}{x}\cdot \left(\frac{O_{x}-O_{\text{base}}}{O_{\text{base}}}\right)$$

(4) $$\varepsilon =1-e^{\left(-\sigma \right)}$$

(5) $$\sigma =\frac{1}{{\overset{‾}{x}}_{obs}}\cdot \sqrt{\sum {\left(x_{obs}-x_{mod}\right)}^{2}}$$

where σ is a normalized error variance between the measured x_obs_ and modeled x_mod_ values normalized to the average of the measured values ${\overset{‾}{x}}_{obs}$.

First, a base run with a parameter setting is modeled (P_base_) to obtain model output values (O_base_). Subsequently, a parameter is disturbed (P_x_, Equation (6)), increasing or decreasing its value by a factor (x), which gives a disturbed output value (O_x_).


(6) $$P_{X}=(1+x)\cdot P_{\text{base}}$$

Sensitivity was calculated with the factors 0.1 and 0.5 (10% and 50% difference, respectively) according to the disturbance of the calibrated parameters (V_s_, V_r_, K_d_) in addition to the boundary conditions (flow rate(Q)) and total suspended solids (TSS). A positive sensitivity value means that an increase (or decrease) in the disturbed parameter will increase (or decrease) the concentrations of the modeled variable; a negative sensitivity value means that the value of the modeled variable will increase (or decrease) contrary to the setting of the disturbed parameter.

The complexity levels considered in the present study with WASP8 are as follows:

- Complexity 1: conservative transport (no sorption), total and dissolved fractions are transported conservatively without sorption reactions between phases, suspended solids, or sediments;
- Complexity 2: equilibrium sorption, a process represented by the partition coefficient (K_d_), which relates the concentration of the metal(loid) phases and the suspended solids, but no interaction with sediments is considered;
- Complexity 3: sorption equilibrium with sediment interaction.

The utility function (U_m_, Equation (7)) was also applied. This maximizes the search for the “best” model, which represents the field data with the lowest error and provides high predictive performance (low sensitivity) (Lindenschmidt et al. [84]).


(7) $$U_{m}=1-\sqrt{\frac{w_{s}\cdot {\overset{ˆ}{s}}_{\mathrm{total},m}^{2}+w_{\varepsilon }\cdot {\overset{ˆ}{\varepsilon }}_{\mathrm{total},m}^{2}}{w_{s}+w_{\varepsilon }}}$$

where ${\overset{ˆ}{s}}_{\mathrm{total},m}^{2}$ and ${\overset{ˆ}{\varepsilon }}_{\mathrm{total},m}^{2}$ are the sensitivity and error of each model (m) normalized to 1, respectively, and $w_{s}$ and $w_{\varepsilon }$ are weighting factors for sensitivity and error, respectively, both with the same weight, each equal to 1 (to maintain the same importance of the factors).

## 3. Results and Discussion

### 3.1. Hydrogeochemical Characterization

**Figure 3.**
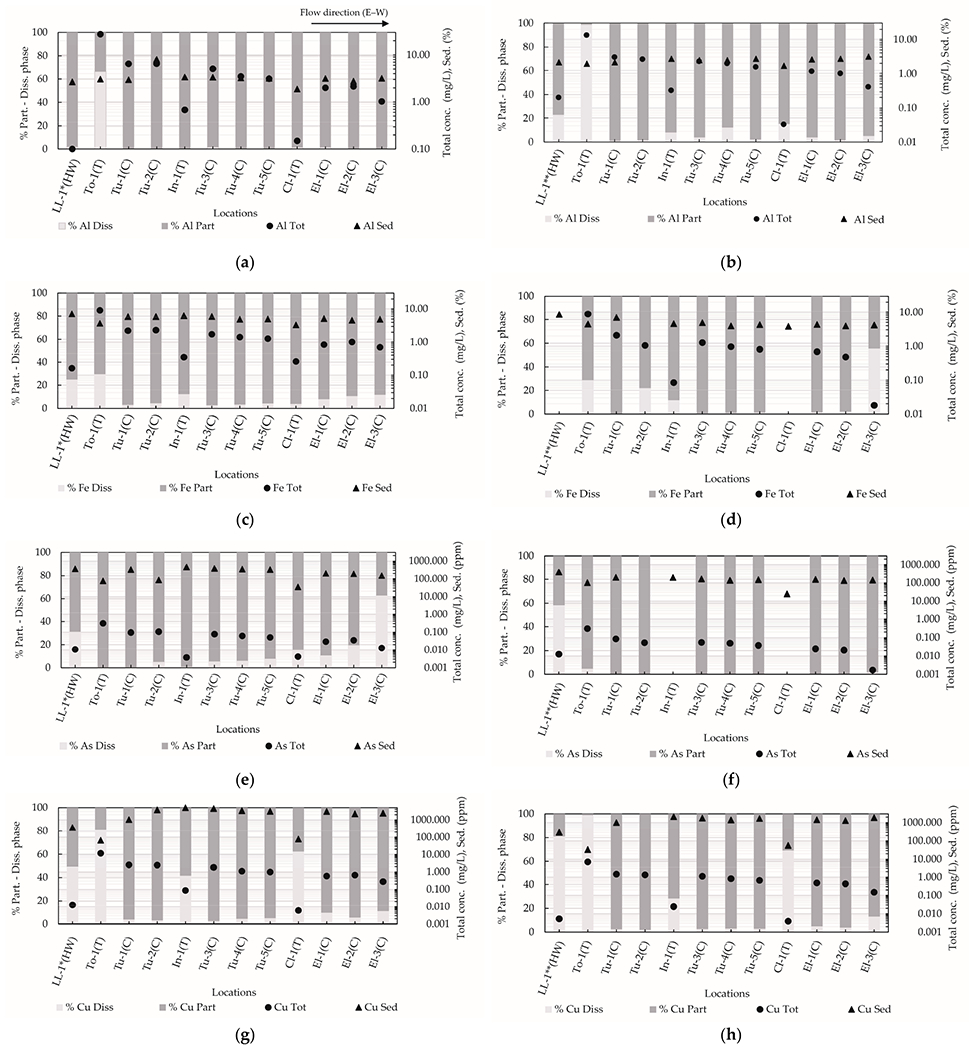
Longitudinal profiles of the dissolved and particulate phase of concentrations of metal(loid)s in the water column (**left axis**) and total concentrations of metal(loid)s in the water column and sediment (**right axis**). (**a**) Al, 2018 campaign; (**b**) Al, 2019 campaign; (**c**) Fe, 2018 campaign; (**d**) Fe, 2019 campaign; (**e**) As, 2018, campaign; (**f**) As, 2019 campaign; (**g**) Cu, 2018 campaign; (**h**) Cu, 2019 campaign. LL-1*: 2018 campaign; LL-1**: 2019 campaign; Diss: dissolved concentration; Part: particulate concentration; Tot: total concentration; Sed: sediment concentration. HW: headwater, initial location of the modeling network; T: tributaries; C: control locations. Notes: 1. Locations without percentages refer to total concentrations reported as below the detection limit; 2. The Tu-2 location, in the 2019 campaign, did not report data on sediment concentration.

Total concentrations were generally higher during September 2018, with a decreasing trend towards downstream locations (Table 2; Supplementary Materials, Table S5). The peaks in the concentrations of constituents are characteristic of acid drainage at the Toro River location (To-1, Figure 3). Likewise, given the acidic pH (average 4.5), it was observed that Al and Cu travel mainly in the dissolved phase (over 60%), while As is mostly in the particulate phase, with a percentage over 95%. In watersheds affected by acid drainage, the total and dissolved concentration of copper may range from 0.5 to 170 mg/L and from 0.03 to 6.0 mg/L, respectively [53]. This range is consistent with the concentration of copper in the Toro River, with slightly higher dissolved concentrations. Subsequently, metal(loid)s were found to travel mainly in the particulate phase downstream of the Toro and La Laguna confluence. This is attributed to the alkaline environment (provided by the alkaline waters of the La Laguna, Incaguaz, and Claro rivers), which, among other factors, favors the precipitation of metal(loid)s.

Streambed sediment concentrations showed an inverse behavior with respect to water concentrations (Figure 3), which was especially clear for Cu downstream of the Claro River (Cl-1) in both campaigns. Additionally, water constituents in the Turbio River were mainly transported as dissolved and suspended load in the water column. As showed a similar pattern to Fe (Figure 3c–f) due to coprecipitation and adsorption to iron oxyhydroxides, which is mostly observed in the Elqui River (El-1-2-3).

### 3.2. Modeling with WASP8

#### 3.2.1. Modeling of Flows, Velocities, and SO_4_^2−^ Concentration

**Figure 4.**
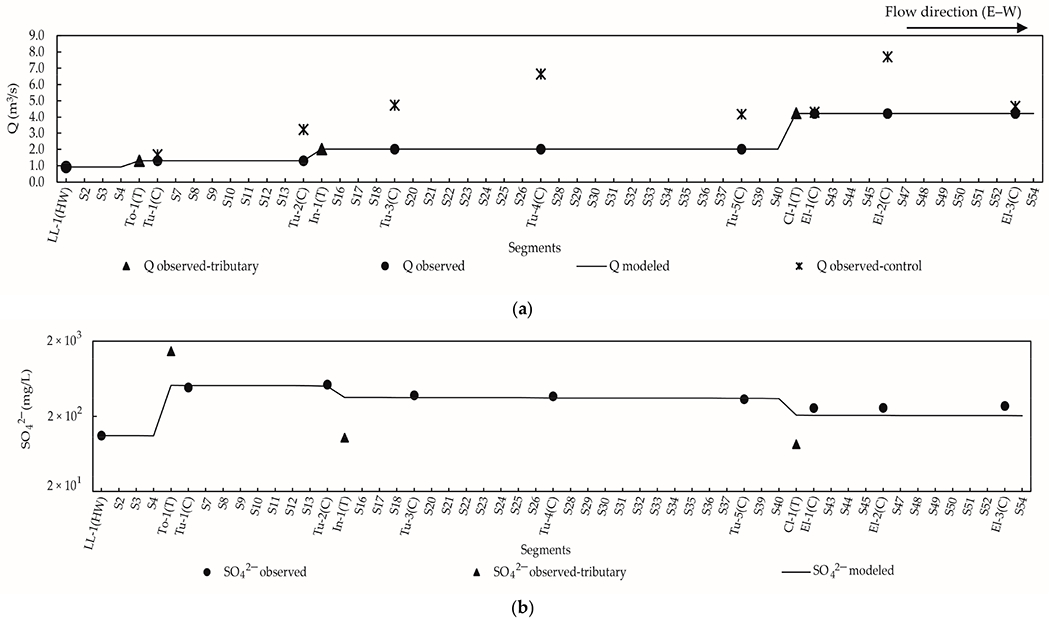
Longitudinal profiles of observed and modeled flows (Q) and sulfates (SO_4_^2−^). (**a**) Flow, 2018 campaign; (**b**) SO_4_^2−^, 2018 campaign. Observed-tributary: data from tributaries; observed-control: data from control locations. HW: headwater, initial location of the modeling network; T: tributaries; C: control locations.

The results show a good fit between the measured and modeled flow rates (Figure 4). Likewise, the indicators reflect good model performance for flow rate (Q) and velocity (U), with classifications from “good” (RRMSE for U) to “very good” for the remaining indicators (see Table 3). This was expected, as the flow rates were the sequential streamflow sum of the tributaries corresponding to the boundary conditions.

On the other hand, when comparing the modeled flows with those estimated from the hydraulic variables, an underestimation was mainly observed in the Turbio River section, from the confluence with the Incaguaz River (In-1) to the Elqui River location in Diaguitas (El-2). This may result from possible diffuse inflows in that reach of the river, as tentative suggested by Rossi et al. [32].

**Figure 5.**
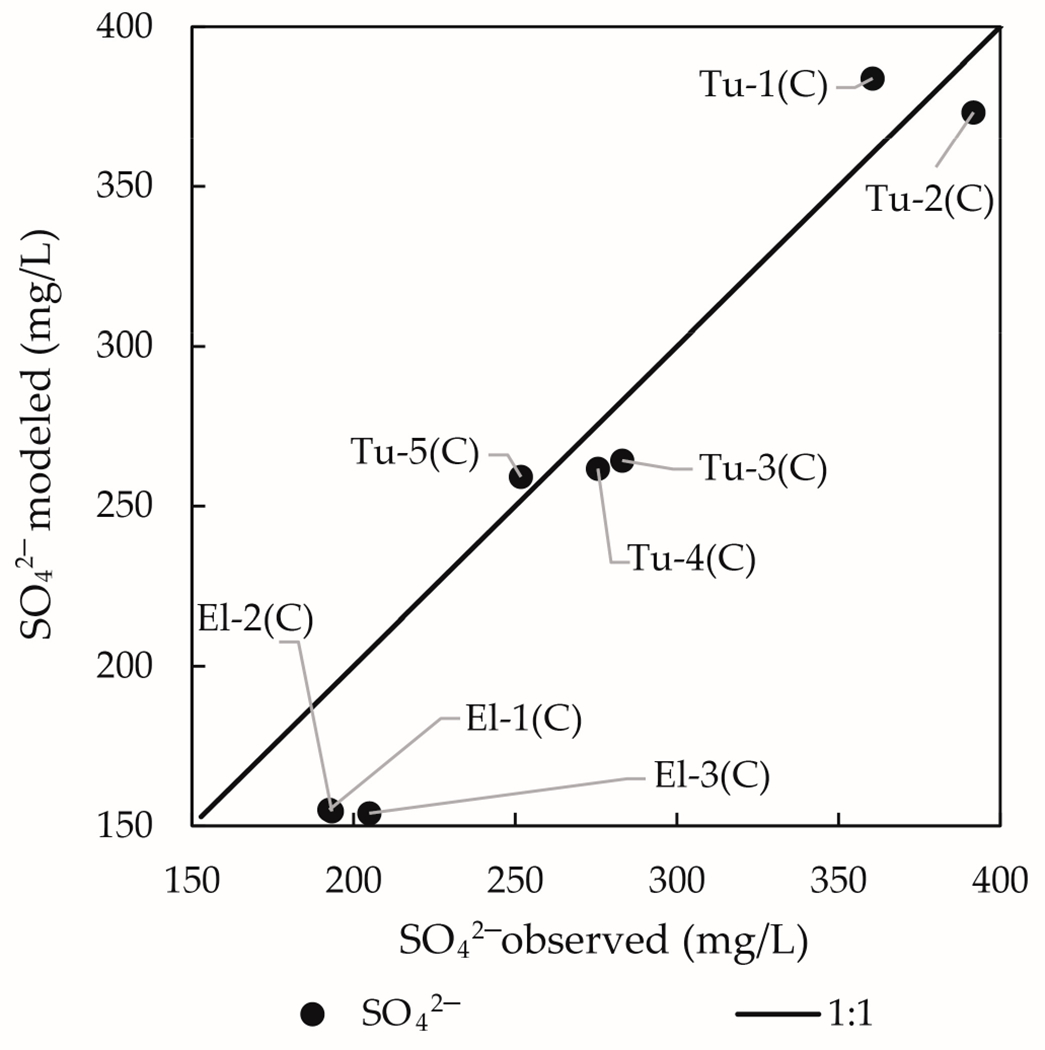
Scatter plot of observed and modeled sulfate (SO_4_^2−^) concentration for control locations. C: control locations.

Figure 5 shows that the modeled SO_4_^2−^ concentrations followed the observed concentrations trend along the network, with some underestimation of the concentrations (except for To-1 and Tu-5 locations), mainly when observed flow rates at control points exceeded modeled values (Figure 4).

The SO_4_^2−^ showed a good fit (Table 3). The observed flow rates (from the sum of the tributaries) were underestimated when compared with those at control locations, which would cause a lack of dilution and a potential overestimation of concentrations. However, as the model underestimated concentrations, generally from the confluence between the Turbio and Incaguaz rivers, it seems unlikely that higher flow rates (from the control locations) were responsible for this underestimation. Therefore, using the flow rates observed from the sum of the tributaries was justified as the model was able to reproduce the behavior of observed flow rates, velocities, and SO_4_^2−^ with a “very good” performance indicator (Table 3).

#### 3.2.2. Modeling of Metal(loid) Concentrations

Model performance for water constituent concentrations was diverse. The performance for the total concentrations showed the following decreasing order: Cu and Al (“very good”), Fe and As (“good”); for dissolved concentrations the performance was as follows: Al and As (“very good”), Cu (“good”) and Fe (“acceptable”) (Table 4). Despite the variability between the different performance indicators, the calibrated model was considered “good” for total and dissolved concentrations.

**Figure 6.**
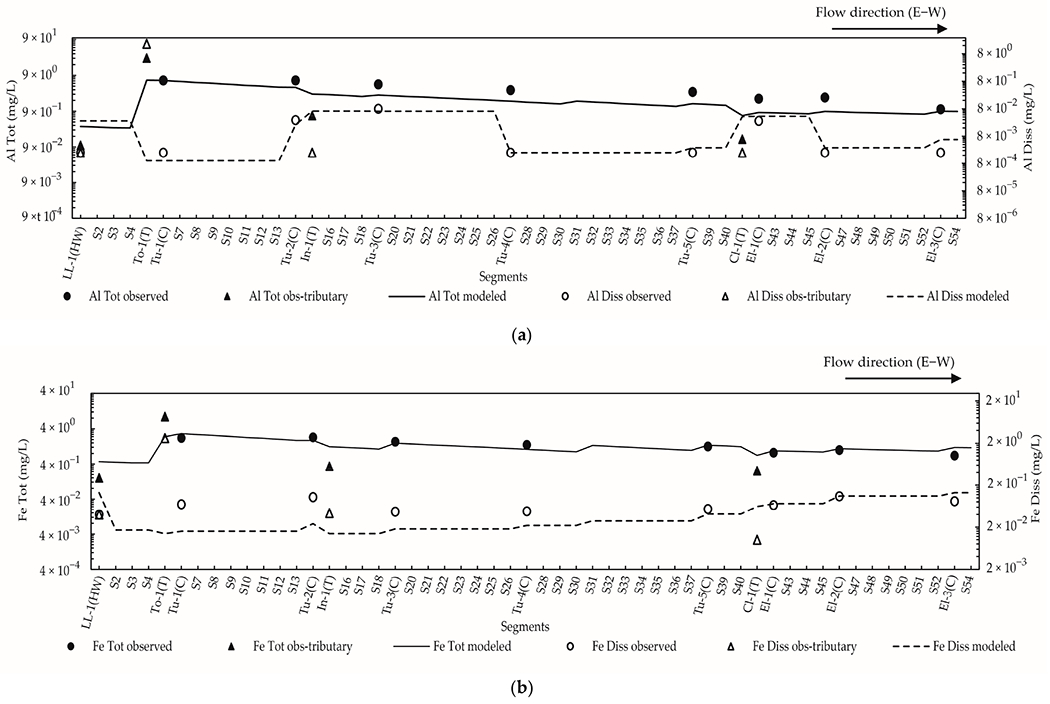
Longitudinal profile of the calibrated model for the constituents in the water column. (**a**) Al concentration and (**b**) Fe concentration. Tot observed: total observed concentration; Tot obs-tributary: total observed concentration in tributaries; Tot modeled: total modeled concentration in control locations; Diss observed: dissolved concentration; Diss obs–tributary: dissolved concentration in tributaries; Diss modeled: dissolved modeled concentration in control locations. HW: headwater, initial location of the modeling network; T: tributaries; C: control locations.

**Figure 7.**
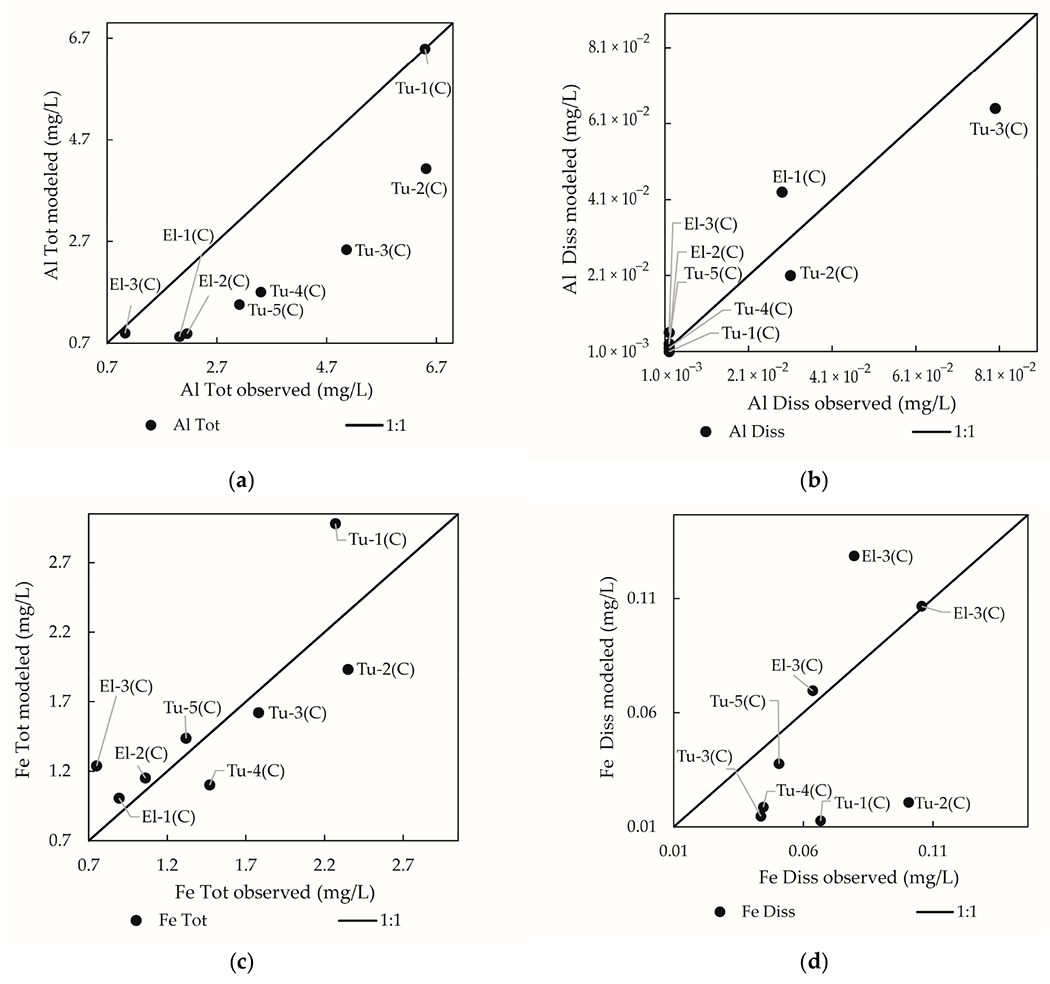
Scatter plots of the calibrated model for observed and modeled constituent concentrations in the water column: (**a**) total Al concentration; (**b**) dissolved Al concentration; (**c**) total Fe concentration; (**d**) dissolved Fe concentration. Tot observed: total observed concentration; Tot modeled: total modeled concentration; Diss observed: dissolved observed concentration; Diss modeled: dissolved modeled concentration. The black line represents the 1:1 ratio. C: control locations.

The model followed the downstream decreasing trend (Figure 6), underestimating total concentrations (Figure 7a,c). The Tu-1 location (after the confluence of the La Laguna and Toro rivers) had a systematic overestimation of total concentrations, except for As. Underestimates occurred systematically from the Tu-2 location; however, model performance improved towards the downstream sections of the Elqui River. On the other hand, the modeled total Fe presented a better fit than the rest of the metal(loid)s, even though it overestimated in the final sections of the modeled network and underestimated the dissolved Fe concentrations for all sections of the Turbio River. Similarly, the sediment indicators were classified as “very good” for all metal(loid)s (Table 4).

As can also be observed in Figure 6, the modeled metal(loid)s remained in the water column (mainly in the upstream and middle zone of the modeled network), which favored their transport along the UWER. This was consistent with the observed data (Figure 3). This can be explained, according to Díaz et al. [51], in that the size of the dominant suspended material contributed by the Toro River was mainly fine (d < 6 μm) and ultrafine (6 < d < 63 μm), which hampers particle coagulation. Consequently, the suspended material and formation of Cu and As-enriched particles downstream of the confluences did not tend to form particles of sufficient size to sediment, as is often the case in other mixed zones affected by acid drainage [53,96]. Additionally, Díaz et al. [51] identified that the dissolved concentrations of Fe and Al were insufficient to favor the coagulation of the particulate metal(loid)s in suspension. This also can explain the stability of Al concentration in the water column (and the other constituents) throughout the basin, except for the El-3 location, where a potential sediment enrichment may have occurred. This condition likely resulted in the favorable performance of the model, especially for Al and Fe, as they are the most complex constituents to address. On the other hand, the WASP8 model does not directly couple precipitation/dilution (with the direct influence of pH and ionic strength), which could generate some deficiencies in modeling the mineral phases of these two constituents [2].

#### 3.2.3. Validation Processes and Performance Indicators

**Figure 8.**
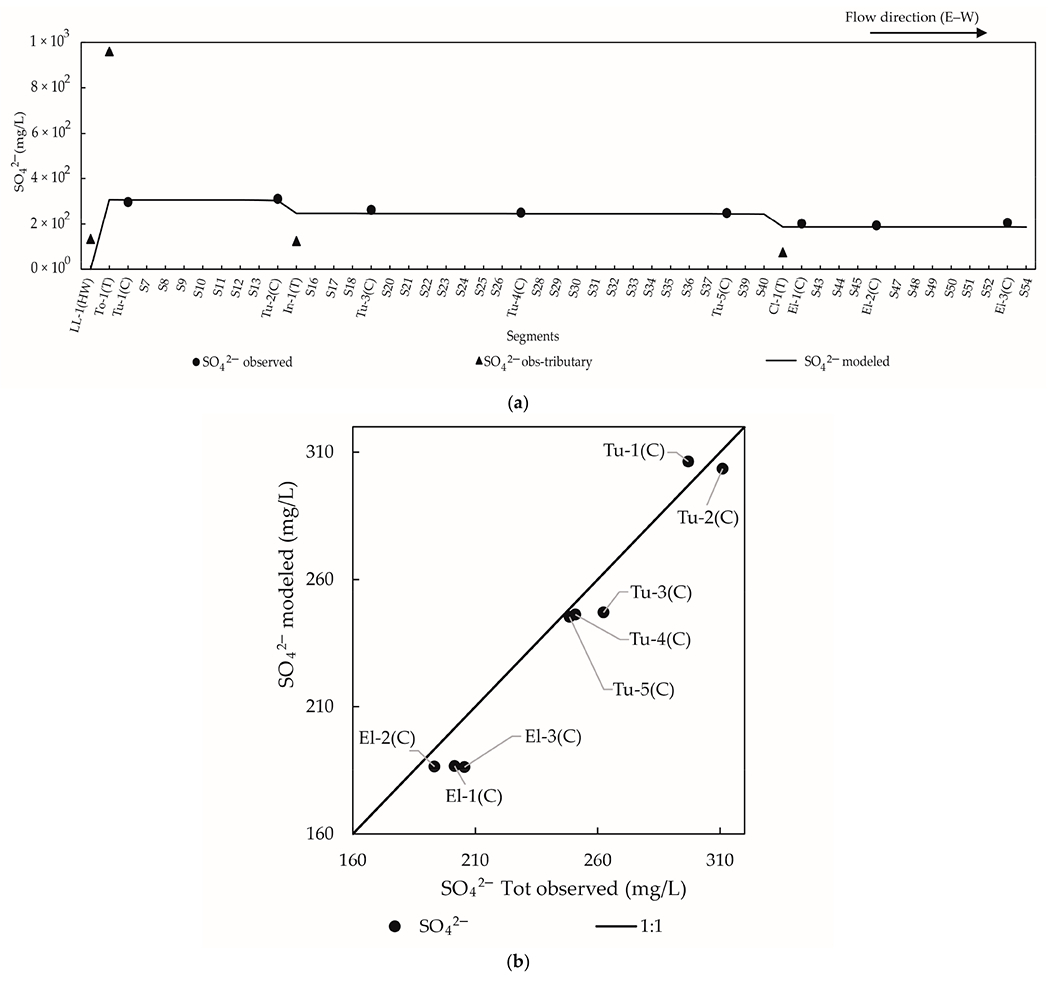
Validation results for sulfate (SO_4_^2−^) concentration. (**a**) Longitudinal profile comparing modeled and observed data; (**b**) scatter plot displaying modeled versus observed data. Obs-tributary: concentration observed in tributaries; mod: modeled concentration. HW: headwater, initial location of the modeling network; T: tributaries; C: control locations.

Regarding the validation of the modeled flows, velocities, and SO_4_^2−^, the model showed good agreement with the observed data throughout the basin, with most indicators classified as “very good” (Table 5, Figure 8).

As seen in Figure 8a, and somewhat similar to what was found in the calibration stage, the adjustment with the SO_4_^2−^ model was consistent with the observed values. Likewise, all of the indicators (Table 5) showed a “very good” performance. The scatter plot (Figure 8b) shows that the model underestimated SO_4_^2−^ concentrations, except at the Tu-1 location, resembling the calibration. Although the flow rates of the 2019 campaign were slightly higher than those of the 2018 campaign, the validation obtained similar classifications for flow, velocity, and SO_4_^2−^ indicators in the “very good” range. Therefore, the use of flow rates estimated from the sum of tributaries was validated.

Concerning the validation for the metal(loid) concentrations, as shown in Table 6, the model was able to satisfactorily represent the total concentrations in the following decreasing order: Cu and Al (“very good”), Fe and As (“good”). Regarding dissolved metal(loid) concentrations, the model obtained a “good” performance classification for Al. However, dissolved Fe, As, and Cu concentrations reported the lowest “insufficient” category. The performance indicators for the sediments presented an overall “very good” ranking, with a slightly lower performance than the calibration results.

**Figure 9.**
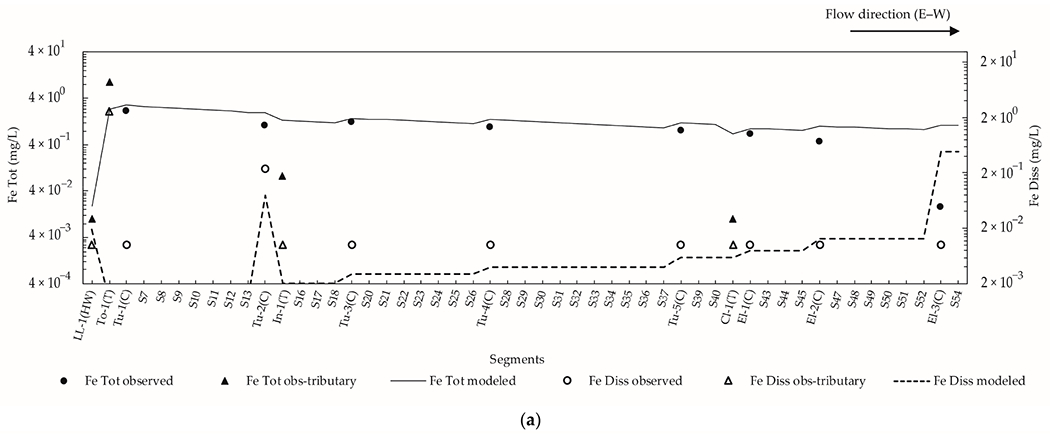

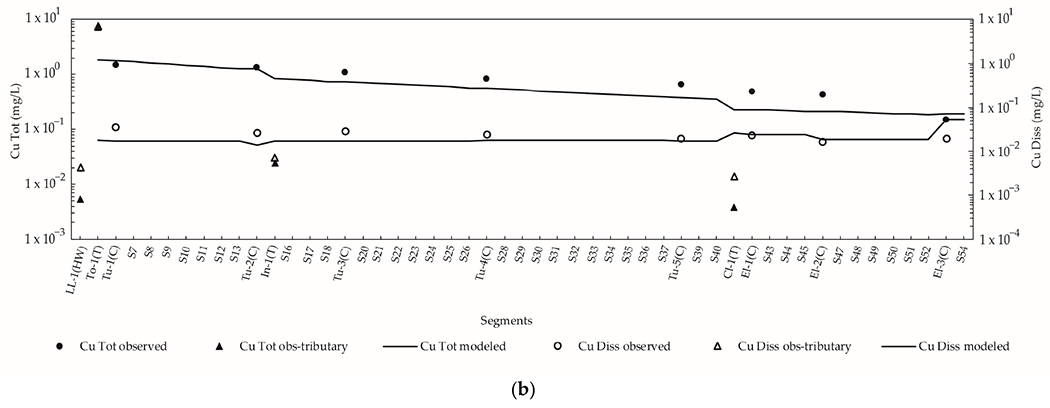
Longitudinal profile of the model validation for the constituents in the water column. (**a**) Fe concentration; (**b**) Cu concentration. Tot observed: total observed concentration; Tot obs-tributary: total observed concentration in tributaries; Tot modeled: total modeled concentration in control locations; Diss observed: dissolved concentration in control locations; Diss obs–tributary: dissolved concentration in tributaries; Diss modeled: dissolved modeled concentration in control locations. HW: headwater, initial location of the modeling network; T: tributaries; C: control locations.

**Figure 10.**
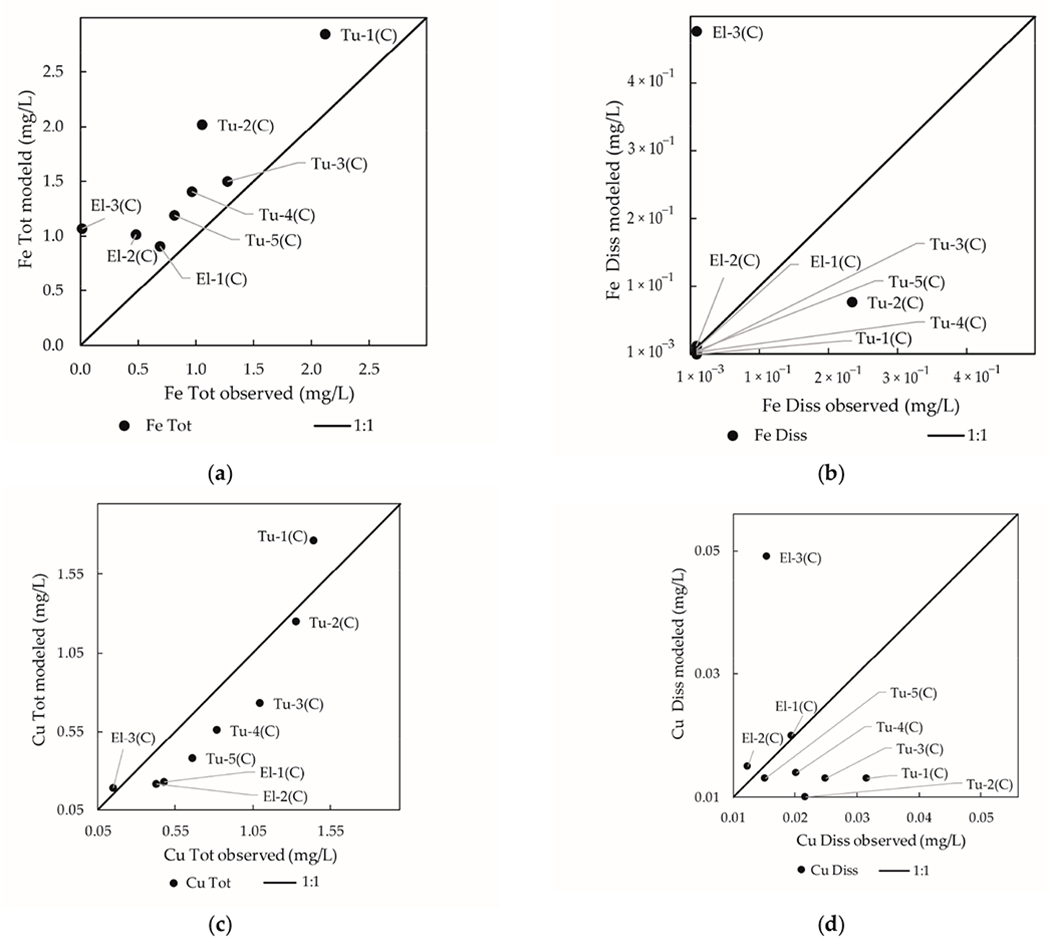
Scatter plots of the model under validation for concentrations of the observed and modeled constituents in the water column and sediments. (**a**) Total Fe concentration; (**b**) dissolved Fe concentration; (**c**) total Cu concentration; (**d**) dissolved Cu concentration. The black line represents the 1:1 ratio. C: control locations.

Figure 9 plots Fe and Cu longitudinal profiles, showing that the validated model followed the metal concentration trend. In general, the validated model underestimated the total and dissolved phases of Al, Cu, and As. Figure 10 shows the scatter plots for Fe and Cu, noting the overestimation of total Fe concentration along the UWER (Figure 10a). However, for the calibration, this only occurred at the beginning and end of the modeled network (Tu-1, Tu-5, and the Elqui River reach). Similar to the calibration, Cu (Figure 10c) was overestimated only at Tu-1 and El-3. On the other hand, the January 2019 campaign reported mainly the dissolved concentrations of Fe and As below the detection limit, making the validation of these two elements quite difficult. It also reported the lowest metal(loid) concentrations and slightly higher flow rates than the September 2018 campaign (Table 2), which was used to calibrate the model. This condition may have contributed to the overall underestimation of Al, As, Cu, and SO_4_^2−^ due to the dilution effect of the total concentrations.

#### 3.2.4. Sensitivity Analysis

**Figure 11.**
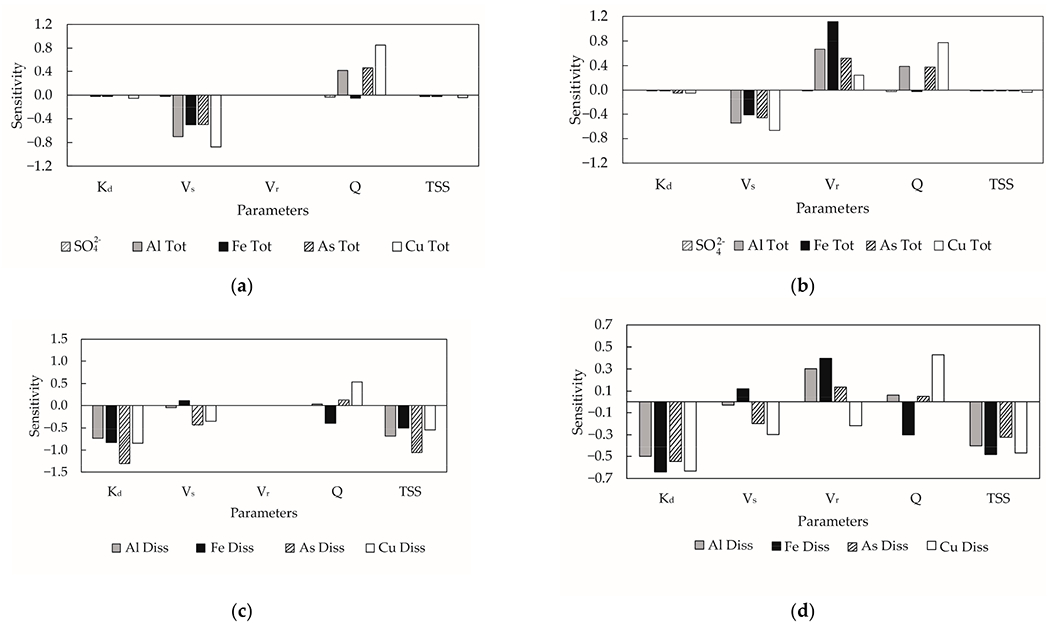
Sensitivity analysis of the calibrated parameters, flow rate (Q), and total suspended solids (TSS) on SO_4_^2−^, Al, Fe, As, and Cu. (**a**) Sensitivity with factor 0.1 for total phase constituent concentration; (**b**) sensitivity with factor 0.5 for total phase constituent concentration; (**c**) sensitivity with factor 0.1 for dissolved phase constituent concentration; (**d**) sensitivity with factor 0.5 for dissolved phase constituent concentration. Tot: total phase concentration; Diss: dissolved phase concentration; K_d_: partitioning coefficient; V_s_: sedimentation velocity; V_r_: resuspended velocity.

As shown in Figure 11a,b, the total concentrations were mainly affected by the disturbances applied to V_s_, V_r_, and Q. When comparing the applied factors, it was observed that the V_r_ parameter did not play an important role with a 0.1-factor disturbance (10% increase), as the initial parameter value (10^−4^ m/d) did not vary sufficiently. However, when the factor 0.5 (50% increase) was applied, the effect of this disturbance on all total metal(loid) concentrations was identified (Figure 11b). As expected, the sensitivity for the V_s_ parameter was negative, meaning that, as this parameter increases, the constituent concentrations decrease. Conversely, the sensitivity for V_r_ was positive, indicating that rising values led to higher concentrations in the water column. SO_4_^2−^ showed a slight variation with the tested Q change, as expected due to its conservative behavior. Likewise, we observed a negative sensitivity for SO_4_^2−^ in the model, i.e., an inverse relationship between flow rates and sulfate concentration, or a dilution behavior (dependency), which agrees with other studies [97,98].

Furthermore, in agreement with Lindenschmidt et al. [84], dissolved concentrations of the constituents (i.e., Al, Fe, As and Cu) were much more sensitive than total concentrations to K_d_ and TSS disturbances. The K_d_ and TSS sensitivity values were negative, which is expected as (among other factors) the higher the K_d_ value, the more the constituents are transported in the particulate phase attached to the TSS [3,99–102]. Additionally, the effect of TSS disturbances was slightly less than that of K_d_. This is because the sorption reactions (and consequently the variation of K_d_) are influenced by several factors besides TSS concentration, such as pH, salinity, redox potential, temperature, concentration, composition, TSS particle size, among others [2,25,28,29,78,87,100,103–105]. On the other hand, K_d_ and TSS disturbances showed similar effects for Fe–Cu and Al–As pairs, demonstrating affinities between constituents. At the lowest disturbance (factor 0.1), As was more sensitive, but Fe and Cu exhibited higher sensitivity as the factor increased, followed by As and Al, showing different responses to disturbances.

**Figure 12.**
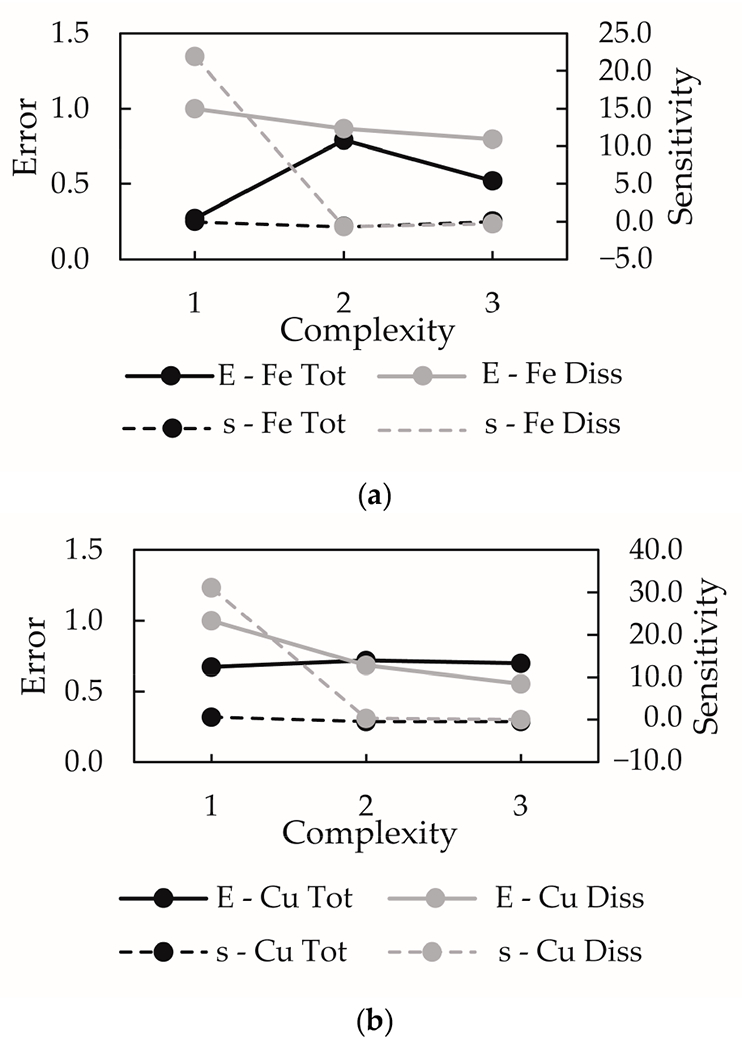
Complexity versus error and sensitivity for Fe and Cu constituents. (**a**) Fe concentration; (**b**) Cu concentration. Tot: total concentration; Diss: dissolved concentration. E: error; s: sensitivity.

Figure 12 shows the error and sensitivity results for total and dissolved Fe and Cu concentrations as a function of model complexity. According to Mbuh et al. [70] and Lindenschmidt et al. [84], complex models can reduce errors, but often at the cost of reduced predictability. This hypothesis was evident for Fe (Figure 12a), where higher model complexity corresponds to lower error and sensitivity, particularly in the transition from model 2 to model 3, with a noticeable decrease in error and a slight increase in sensitivity. Generally, metal(loid) sensitivity decreased between models 1 and 2, then increased slightly between models 2 and 3. No clear pattern was observed for copper (Figure 12b). The error for total Cu concentration remained at 0.7, while for dissolved Cu it decreased from 1.0 to 0.6 with increasing complexity. Regarding sensitivity, the total concentration increased slightly from −0.5 (model 2) to −0.4 (model 3), while the dissolved concentration decreased from 31 to 0.

**Figure 13.**
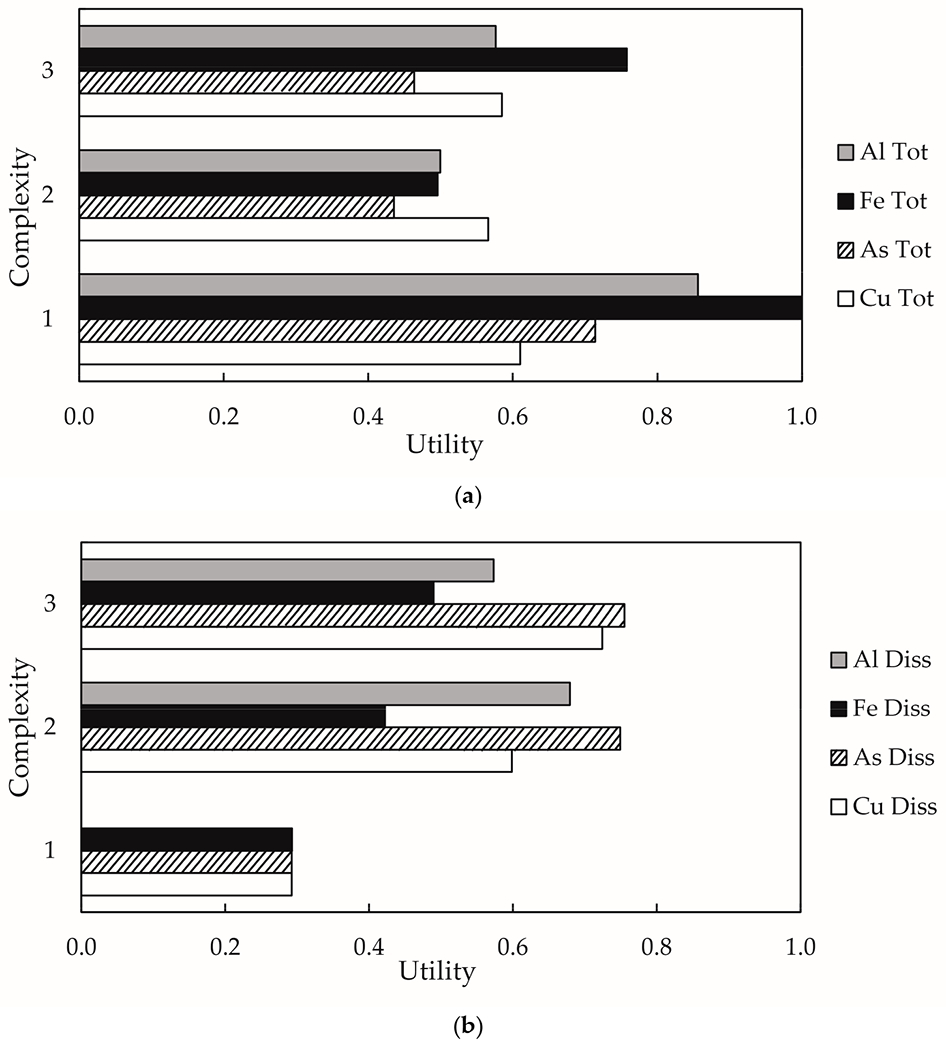
Utility index vs. complexity model for total and dissolved metal(loid)s concentration. (**a**) Total concentration and (**b**) dissolved concentration.

Figure 13 shows utility indicator results as a function of model complexity. Model 1 (Figure 13a) generally performed the “best” utility for total concentrations and the “worst” for dissolved concentrations (Figure 13b). This is because, when sorption is not considered or the values of K_d_ are very low, e.g., less than 10^3^ L/kg, the constituent is assumed to be mainly transported in dissolved or ionic form [99,101,102,105]. Therefore, model 1 showed the dissolved concentrations as equal to the total concentrations, reducing the representation of the dissolved fraction. On the other hand, in most of the study area, metal(loid)s were found to be transported in the particulate phase in the modeled main network (without tributaries) (see Figure 3 and Table 2). For this reason, data on total, dissolved, or particulate concentrations are essential to avoid interpretation errors in model results.

Model 3 was more useful for the metal(loid)s as it balanced total and dissolved concentrations results. Therefore, not only did the sorption process become dominant in the fate and transport of metal(loid)s, but the interaction with sediments also played an essential role in the behavior of metal(loid)s in the study area. However, especially for the dissolved Al concentration, model 2 showed a “better” utility indicator than model 3, demonstrating the different behavior among the constituents.

## 4. Conclusions

Using observations and mechanistic modeling, this work suggests how the Toro River metal(loid) concentrations are elevated by acid drainage inputs. Concentrations decreased downstream of the La Laguna River confluence, with metal(loids) transported in the particulate phase, remaining suspended until the Claro River, where a decline in concentrations suggested potential sediment enrichment. Calibrated WASP8 model parameters, including sedimentation velocity, resuspension velocity, and partition coefficient, were consistent with the expected ranges and the WASP8 model accurately captured trends in metal(loid) concentrations in the upper Elqui River basin. Sensitivity analysis showed that total concentrations were sensitive to disturbances in sedimentation velocity, resuspension velocity, and flow rate values, while dissolved concentrations were affected by disturbances in partition coefficient and total suspended solids values.

With limited data, this study developed a simplified conceptual model of the dominant metal(loid) transport processes in the upper Elqui River basin. During calibration, validation, and sensitivity testing, the model could continuously follow trends and spatially represent constituent concentrations, contrasting with a discrete balance based on total average concentrations. This work highlights opportunities for improvement, such as the consideration of other geochemical processes (i.e., precipitation/dissolution and oxidation-reduction), use of a zone-differentiated partition coefficient, and keeping records of total suspended solids concentration at the sampled locations. These enhancements would increase the accuracy of concentration estimates, especially for Fe, since the sorption process may not be sufficient to describe Fe behavior, and it may be necessary to include oxidation reactions.

Future studies should attempt to calibrate a partition coefficient based on sectorspecific factors, including variations in pH, total suspended solids concentrations, and the diameter of suspended particles in the water column. Additionally, the transport of solids to and from the sediments should be studied more, particularly during erosive events and high flows. Finally, to enhance the spatial representativeness of the study, it is necessary to increase the number of sampling locations, particularly in the middle and lower reaches of the Turbio River, as well as to increase the number of campaigns to represent the seasonal variability of the behavior of metal(loid)s and calibrated parameters values.

## Supplementary Material

Supplement1

The following supporting information can be downloaded at https://www.mdpi.com/article/10.3390/w17131905/s1. Please refer to [105–108] for the relevant references.

## Figures and Tables

**Table 1.** Interpretation of indicator results categorization.

Category	RRMSE (%)	R^2^	d
Very good	RRMSE ≤ 19	0.80–1.00	0.80–1.00
Good	20 ≤ RRMSE ≤ 49	0.60–0.79	0.60–0.79
Acceptable	50 ≤ RRMSE ≤ 79	0.40–0.59	0.40–0.59
Poor	80 ≤ RRMSE ≤ 100	0.20–0.39	0.20–0.39
Insufficient	>100	0.00–0.19	0.00–0.19

Note: Source: own elaboration based on the bibliographic review [1,2,8,69,80,83,91–95]. RRMSE: relative root mean square error; R^2^: coefficient of determination; d: index of agreement.

**Table 2.** Summary table of water constituent concentrations, pH, and flow rates for each sampling location.

Locations	Al Tot (mg/L)	Al Diss (mg/L)	Fe Tot (mg/L)	Fe Diss (mg/L)	As Tot (μg/L)	As Diss (μg/L)	Cu Tot (mg/L)	Cu Diss (mg/L)	SO_4_^2−^ (mg/L)	pH	Q obs (m^3^/s)
Ll-1 (2018) (HW)	0.10	<0.002	0.16	0.04	10.63	3.32	0.013	0.006	83.1	8.13	0.92
Ll-1 (2019) (HW)	0.20	0.046	<0.01	<0.01	12.60	7.35	0.005	0.004	134.1	8.20	1.07
Avg. La Laguna River (n = 2)	0.15	0.024	0.08	0.02	11.62	5.34	0.009	0.005	108.6	8.17	1.00
To-1 (2018) (T)	27.54	18.300	8.98	2.65	305.82	<0.03	11.850	9.620	1099.0	4.50	0.38
To-1 (2019) (T)	13.69	13.524	8.90	2.57	305.50	14.24	7.111	7.036	958.0	4.43	0.50
Avg. Toro River (n = 2)	20.62	15.912	8.94	2.61	305.66	7.13	9.481	8.328	1028.5	4.47	0.44
Tu-1 (2018) (C)	6.49	<0.002	2.22	0.07	94.84	0.32	2.550	0.095	360.4	7.58	1.67
Tu-1 (2019) (C)	3.03	0.031	2.12	<0.01	84.16	<0.03	1.442	0.036	297.0	7.70	3.22
Tu-2 (2018) (C)	6.51	0.031	2.30	0.10	105.50	5.21	2.490	0.076	391.6	7.86	3.22
Tu-2 (2019) (C)	2.63	0.033	1.06	0.24	52.39	<0.03	1.330	0.026	311.0	8.16	2.10
Tu-3 (2018) (C)	5.06	0.080	1.73	0.05	78.52	4.24	1.850	0.044	283.0	7.99	4.74
Tu-3 (2018) (C)	2.29	0.082	1.28	<0.01	54.00	<0.03	1.099	0.029	262.4	8.12	4.78
Tu-4 (2018) (C)	3.50	<0.002	1.42	0.05	62.38	3.63	1.120	0.049	275.6	7.95	6.64
Tu-4 (2019) (C)	1.94	0.241	0.97	<0.01	49.00	<0.03	0.819	0.024	250.9	8.06	3.00
Tu-5 (2018) (C)	3.11	<0.002	1.27	0.05	51.15	3.91	1.000	0.049	251.7	7.91	4.17
Tu-5 (2019) (C)	1.60	0.034	0.82	<0.01	37.54	<0.03	0.664	0.019	248.5	7.91	2.81
Avg. Turbio River (n = 10)	3.62	0.054	1.52	0.06	66.95	1.74	1.436	0.045	293.2	7.92	3.64
In-1 (2018) (T)	0.68	<0.002	0.35	0.04	3.75	<0.03	0.088	0.037	78.1	7.52	0.72
In-1 (2019) (T)	0.32	0.025	0.09	<0.01	<0.03	<0.03	0.025	0.007	124.2	8.15	0.70
Avg. Incaguaz River (n = 2)	0.50	0.01	0.22	0.02	1.88	<0.03	0.057	0.022	101.2	7.84	0.71
Cl-1 (2018) (T)	0.15	<0.002	0.26	<0.01	4.16	0.64	0.006	0.004	64.1	7.74	2.20
Cl-1 (2019) (T)	0.03	0.005	<0.01	<0.01	<0.03	<0.03	0.004	0.003	75.7	7.97	1.17
Avg. Claro River (n = 2)	0.09	0.003	0.13	<0.01	2.09	0.33	0.005	0.003	69.9	7.86	1.69
El-1 (2018) (C)	2.02	0.029	0.84	0.07	29.24	3.12	0.579	0.056	192.5	8.19	4.31
El-1 (2019) (C)	1.20	0.048	0.69	<0.01	24.30	<0.03	0.480	0.024	201.6	8.08	4.73
El-2 (2018) (C)	2.16	<0.002	1.01	0.11	34.93	6.84	0.660	0.036	193.3	8.18	7.71
El-2 (2019) (C)	1.02	0.017	0.48	<0.01	20.60	<0.03	0.427	0.017	193.3	8.30	2.34
El-3 (2018) (C)	1.03	<0.002	0.70	0.08	12.70	7.97	0.278	0.031	204.9	7.81	4.66
El-3 (2019) (C)	0.42	0.022	0.02	<0.01	1.72	<0.03	0.149	0.020	205.6	7.91	5.40
Avg. Elqui River (n = 6)	1.31	0.020	0.62	0.05	20.58	3.00	0.429	0.030	198.5	8.08	4.86
Avg. UWER (n = 24)	3.61	1.357	1.57	0.26	59.81	2.54	1.502	0.723	280.8	7.68	3.05

Note: Tot: total concentration; Diss: dissolved concentration; Avg.: average; Avg. UWER: average for all samples in the upper Elqui River watershed; n: number of locations considered. Q obs.: observed flow rate. HW: headwater, initial location of the modeling network; T: tributaries; C: control locations.

**Table 3.** Indicator results for Q, U, and SO_4_^2−^—calibration of the 2018 campaign.

Indicator	Q	U	SO_4_^2−^
RRMSE (%)	0.0	0.0	10.9
R^2^	1.0	1.0	0.9
D	1.0	1.0	0.9

Note: Q: flow rate; U: velocity; RRMSE: relative root mean square error; R^2^: coefficient of determination; D: index of agreement.

**Table 4.** Indicator results for constituents—calibration of the 2018 campaign.

Indicators	Water Column								Sediments			

	Al		Fe		As		Cu		Al	Fe	As	Cu

	Tot	Diss	Tot	Diss	Tot	Diss	Tot	Diss				
RRMSE (%)	43.5	43.8	25.9	56.3	61.4	26.2	42.4	28.6	3.2	3.4	3.1	6.4
R^2^	0.8	0.9	0.7	0.3	0.7	0.9	0.9	0.4	1.0	1.0	1.0	0.9
d	0.8	0.9	0.9	0.6	0.7	0.9	0.9	0.7	1.0	0.9	1.0	0.9

Note: Tot: total concentration; Diss: dissolved concentration. RRMSE: relative root mean square error; R^2^: coefficient of determination; d: index of agreement.

**Table 5.** Indicator results for Q, U, and SO_4_^2−^—validation of the calibrated model for the 2019 campaign.

Indicators	Q	U	SO_4_^2−^
RRMSE (%)	0.0	0.0	4.7
R^2^	1.0	1.0	0.9
d	1.0	1.0	0.9

Note: Q: flow rate; U: velocity; RRMSE: relative root mean square error; R^2^: coefficient of determination; d: index of agreement.

**Table 6.** Indicator results for constituents—validation of the calibrated model for the 2019 campaign.

Indicators	Water Column								Sediments			

	Al		Fe		As		Cu		Al	Fe	As	Cu

	Tot	Diss	Tot	Diss	Tot	Diss	Tot	Diss				
RRMSE (%)	24.1	63.4	68.9	457.9	42.5	1146.9	31.3	62.1	4.6	5.2	3.3	12.2
R^2^	0.8	0.7	0.8	0.0	0.8	0.3	0.9	0.1	0.9	1.0	0.9	0.8
d	0.9	0.9	0.8	0.2	0.9	0.0	0.9	0.1	0.9	0.9	0.9	0.9

Note: Tot: total concentration; Diss: dissolved concentration. RRMSE: relative root mean square error; R^2^: coefficient of determination; d: index of agreement.

## Data Availability

The raw data supporting the conclusions of this article will be made available by the authors upon request.

## Abbreviations

The following abbreviations are used in this manuscript:

A: Cross-sectional area
ARD: Acid rock drainage
C: Metal(loid) concentration
C_d_: Dissolved concentration
Cl-1(T): Claro River (tributary)
Cp: Particulate concentration
D: Depth of the cross-sectional area of the stream
d: Index of agreement
D_L_: Longitudinal dispersion coefficient
El-1(C): Elqui River at Albarrobal location (control location)
El-2(C): Elqui River at Diaguitas location (control location)
El-3(C): Elqui River at Gualliguaica location (control location)
ENSO: Niño—southern oscillation
HW: Headwater location
In-1(T): Incaguaz River location (tributary)
K_d_: Partition coefficient
L: Model segment length
Ll-1(HW): La Laguna River location (headwater)
(T): Tributary location
(C): Control location for model performance evaluation
OTIS: One-dimensional transport with inflow and storage
O_x_: Disturbed output value for sensitivity index
P_base_: Base parameter value for sensitivity index
PDO: Pacific decadal oscillation
PHREEQC: pH redox equilibrium model
P_x_: Disturbed parameter value for sensitivity index
Q: Flow rate
R^2^: Coefficient of determination
RRMSE: Relative root mean square error
s: Sensitivity index
${\overset{ˆ}{s}}_{\mathrm{total},m}^{2}$: Model sensitivity for complexity function
S_K_: Total kinetic transformation rate
S_L_: Direct or diffuse loading rate
SR: Stream routine
To-1(T): Toro River location (tributary)
TSS: Total suspended solids
Tu-1(C): Turbio River after the confluence with the La Laguna River location (control location)
Tu-2(C): Turbio River before the confluence with the Incaguaz River location (control location)
Tu-3(C): Turbio River at Balala location (control location)
Tu-4(C): Turbio River at Huanta location (control location)
Tu-5(C): Turbio River at Varillar location (control location)
U: Flow rate velocity
U_m_: Utility function
UWER: Upper Watershed of the Elqui River
U_x_: Longitudinal advective velocity
V: Segment volume
V_r_: Resuspension velocity
V_s_: Sedimentation velocity
W: Width
WASP: Water quality analysis simulation program
WASP8: Water quality analysis simulation program version 8
$w_{s}$: Weighting factor for utility function
$w_{\varepsilon }$: Error factor for utility function
x: Factor of disturbance for sensitivity index
${\overset{‾}{x}}_{\text{obs}}$: Average of the measured values
x_mod_: Modeled value normalized to the average for sensitivity index
x_obs_: Measurement value normalized to the average for sensitivity index
ε: Error for sensitivity index
${\overset{ˆ}{s}}_{\mathrm{total},m}^{2}$: Error model for utility function
σ: Normalized error variance for sensitivity index
