# Supplementary material for: Modeling Metal(loid)s Transport in Arid Mountain Headwater Andean Basin: A WASP-Based Approach
Source: Water (Basel). Author manuscript; Available in PMC 2026 Jun 26. (PMC12292266; doi:10.3390/w17131905)
Supplement: Supplement1 [file NIHMS2094958-supplement-Supplement1.zip › water-3635594-supplementary.pdf]

Supplementary Materials

S1. Complementary pictures of the different locations along the Upper Watershed of the Elqui River

The following images are presented for each sampling location along the UWER.

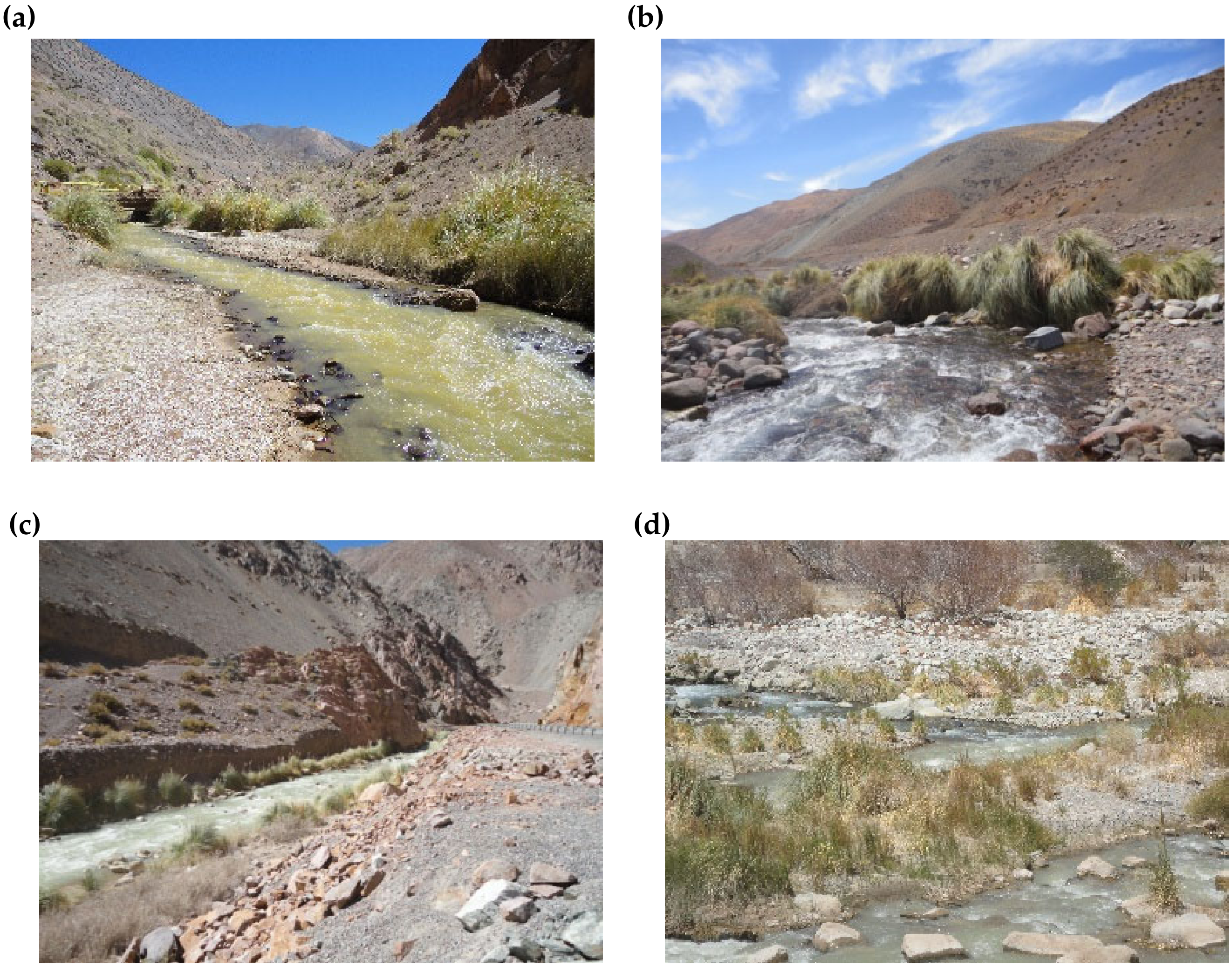

Figure S1. Pictures of the different locations along the Upper Watershed of the Elqui River. (**a**) LL-1(HW), La Laguna River; (**b**) To-1(T), Toro River; (**c**) Tu-1(C), Turbio River after the confluence with La Laguna River; (**d**) Tu-2(C), Turbio River before the confluence with the Incaguaz River; (**e**) In-1(T), Incaguaz River; (f) Tu-3(C), Turbio River at Balala; (**g**) Tu-4(C), Turbio River at Huanta; (**h**) Tu-5(C), Turbio River at Varillar; (**i**) Cl-1(T), Claro River; (**j**) El-1(C), Elqui River at Algarrobal ; (**k**) El-2(C), Elqui River at Diaguitas; (**l**) El-3(C), Elqui River at Gualliguaica.

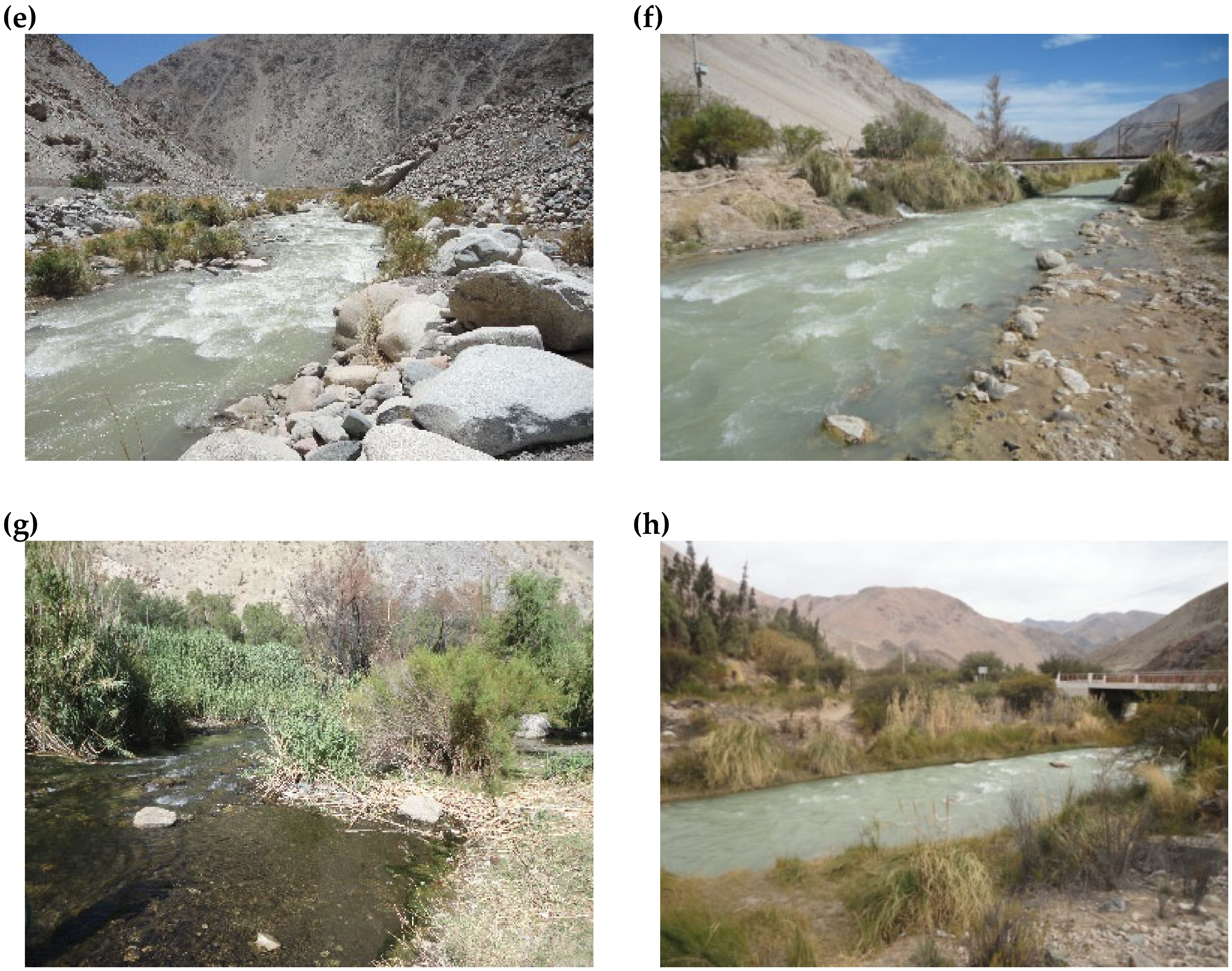

Figure S1 continuation. Pictures of the different locations along the Upper Watershed of the Elqui River. (**a**) LL-1(HW), La Laguna River; (**b**) To-1(T), Toro River; (**c**) Tu-1(C), Turbio River after the confluence with La Laguna River; (**d**) Tu-2(C), Turbio River before the confluence with the Incaguaz River; (**e**) In-1(T), Incaguaz River; (**f**) Tu-3(C), Turbio River at Balala; (**g**) Tu-4(C), Turbio River at Huanta; (**h**) Tu-5(C), Turbio River at Varillar; (**i**) Cl-1(T), Claro River; (**j**) El-1(C), Elqui River at Algarrobal ; (**k**) El-2(C), Elqui River at Diaguitas; (**l**) El-3(C), Elqui River at Gualliguaica.

(i)

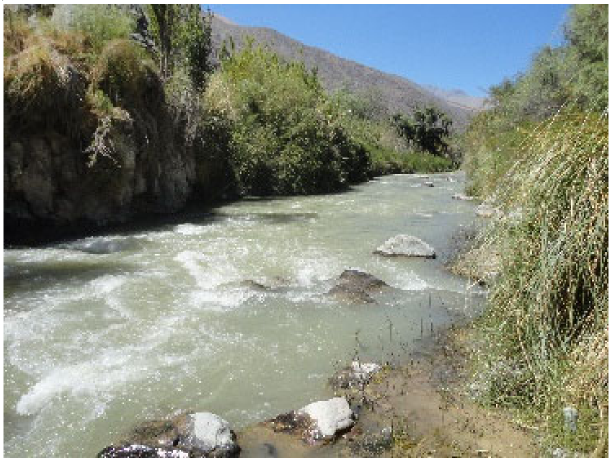

(j)

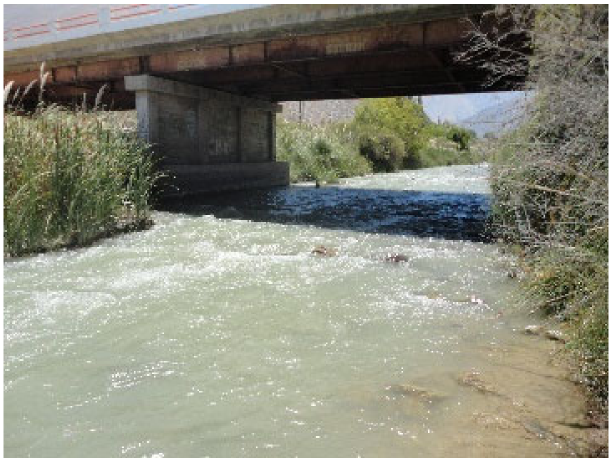

(k)

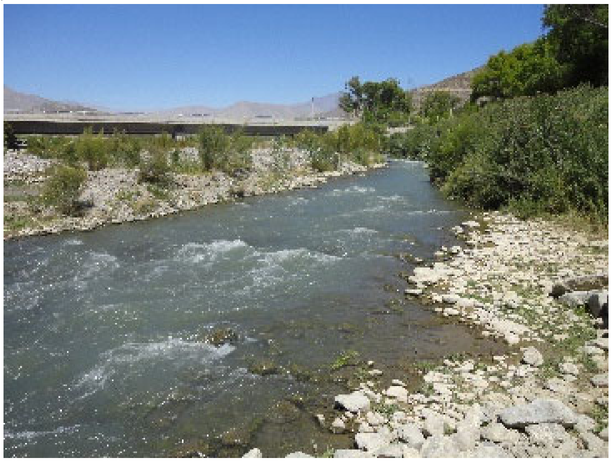

Figure S1 continuation. Pictures of the different locations along the Upper Watershed of the Elqui River. (**a**) LL-1(HW), La Laguna River; (**b**) To-1(T), Toro River; (**c**) Tu-1(C), Turbio River after the confluence with La Laguna River; (**d**) Tu-2(C), Turbio River before the confluence with the Incaguaz River; (**e**) In-1(T), Incaguaz River; (**f**) Tu-3(C), Turbio River at Balala; (**g**) Tu-4(C), Turbio River at Huanta; (**h**) Tu-5(C), Turbio River at Varillar; (**i**) Cl-1(T), Claro River; (**j**) El-1(C), Elqui River at Algarrobal ; (**k**) El-2(C), Elqui River at Diaguitas; (**l**) El-3(C), Elqui River at Gualliguaica.

S2. Estimated total suspended solids (TSS) for the 2018 and 2019 campaigns

This section presents the Total Suspended Solids (TSS) estimation results for the 2018 and 2019 campaigns based on data from the 2020 campaign. As shown in the Table S1, TSS measurements were not available for Claro River; instead, data from Incaguaz River were used based on the similarity of physicochemical parameters and flow rates.

Table S1. Estimated total suspended solids (TSS) for the 2018 and 2019 campaigns from the 2020 campaign.

Puntos	2020 Campaign		2019 Campaign		2018 Campaign	
	Turbidity (NTU)	TSS observed (mg/L)	Turbidity (NTU)	TSS Estimated (mg/L)	Turbidity (NTU)	TSS Estimated (mg/L)
Toro River before La Laguna River	107.0	114.5	122.0	110.2	65.0	61.1
La Laguna River before Toro River	13.1	11.1	20.7	19.1	2.0	1.6
Incaguaz River before Turbio River	5.0	3.3	5.0	2.9	4.3	2.5
Claro River before Turbio River	N.d.	N.d.	1.6	1.0	2.5	1.5

N.d.: No data.

S3. Verification of Steady State

Figures S2 shows how the concentrations of the metal(loid)s change over time until a steady state is reached. The start date of the modeling was 09/25/2018. Final data were retrieved when a steady state was reached, which is when the difference in concentrations of all constituents between two dates was zero (see Table S4). The data retrieval date was 10/15/2018.

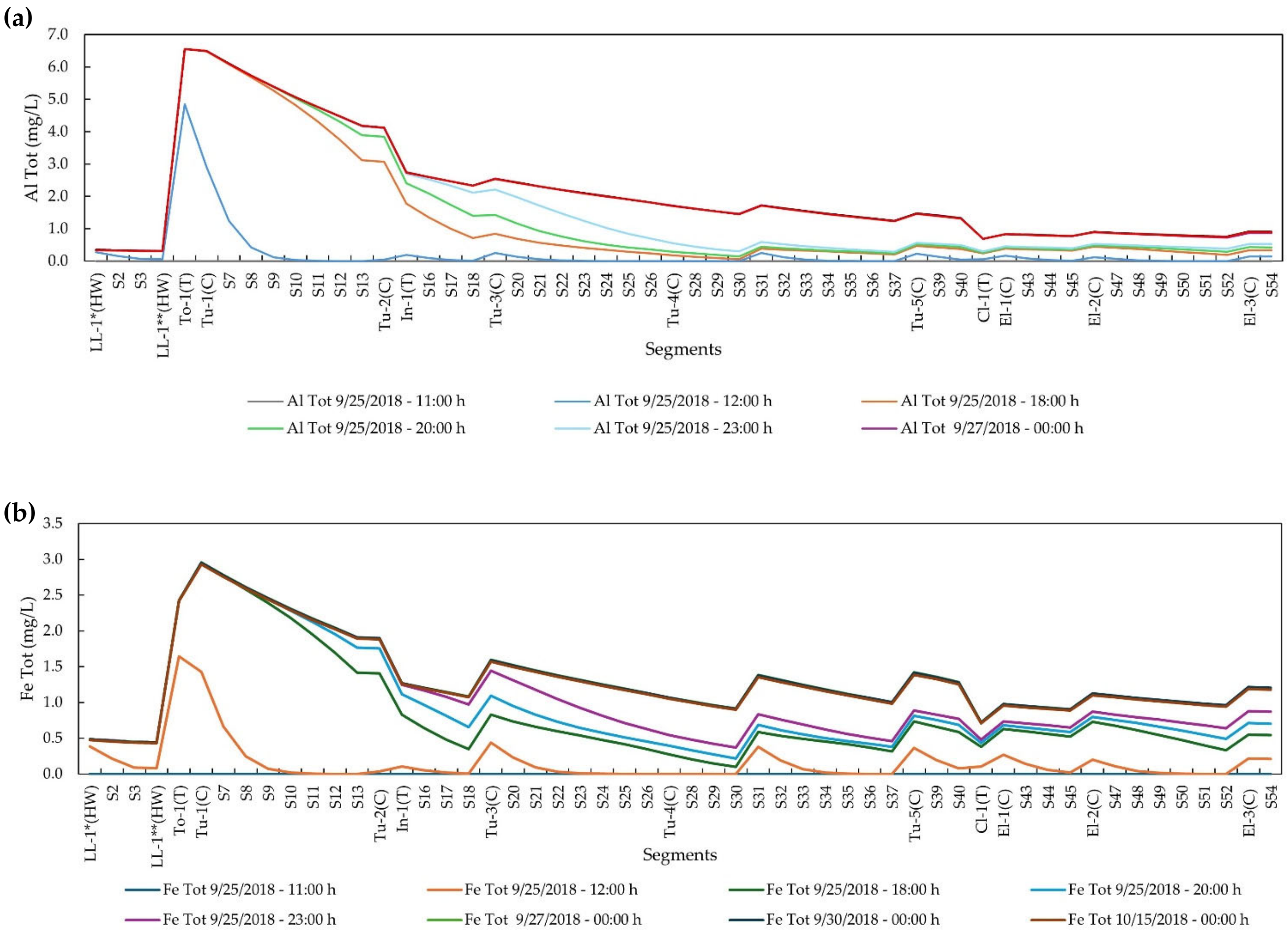

Figure S2. Verification of the steady state achieved. (**a**) Al concentration and (**b**) Fe concentration in the water column. HW: Headwater, initial location of the modeling network; T: tributaries; C: control locations

(a)

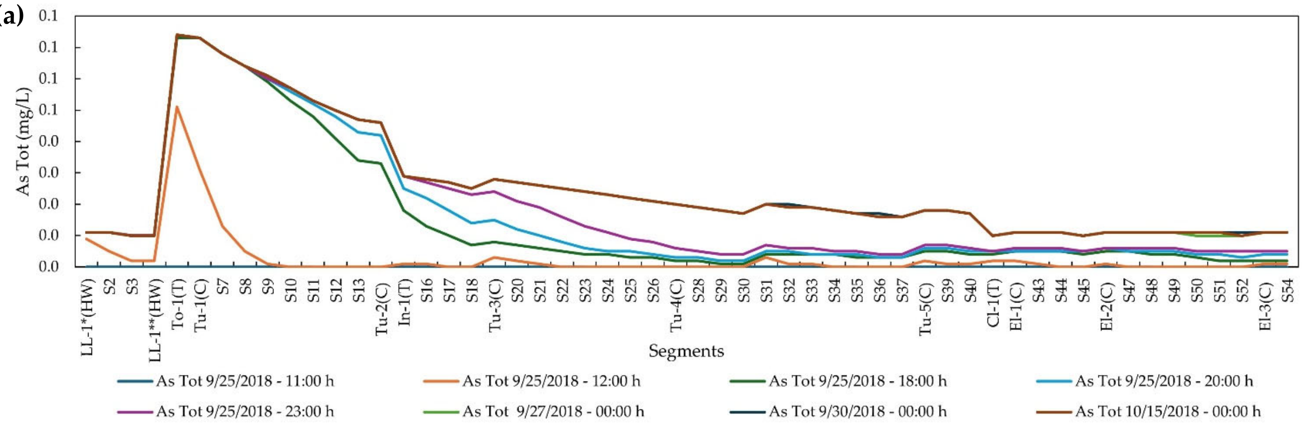

(b)

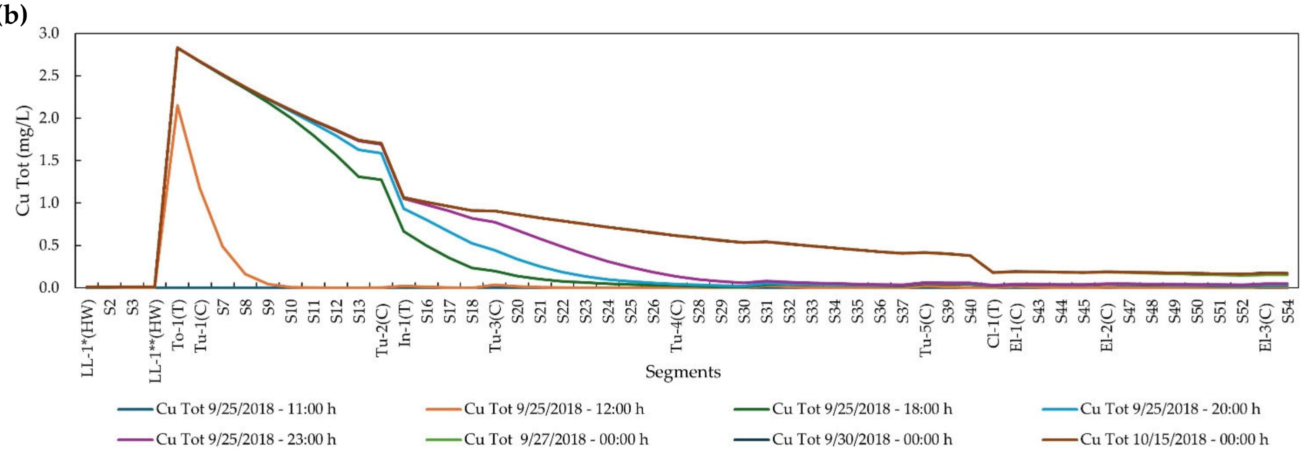

Figure S2 continuation. Verification of the steady state achieved. (**a**) As concentration and (**b**) Cu concentration in the water column. HW: Headwater, initial location of the modeling network; T: tributaries; C: control locations

Table S2. Differences in total constituent concentration by date and time of WASP8 modeling.

Locations	Al Tot (mg/L)		Fe Tot (mg/L)		As Tot (mg/L)		Cu Tot (mg/L)	
	(10/15/2018, 00:00) - (9/30/2018, 00:00 h)	(10/15/2018, 00:00) - (9/25/2018, 12:00 h)	(10/15/2018, 00:00) - (9/30/2018, 00:00 h)	(10/15/2018, 00:00) - (9/25/2018, 12:00 h)	(10/15/2018, 00:00) - (9/30/2018, 00:00 h)	(10/15/2018, 00:00) - (9/25/2018, 12:00 h)	(10/15/2018, 00:00) - (9/30/2018, 00:00 h)	(10/15/2018, 00:00) - (9/25/2018, 12:00 h)
LL-1(HW)	0.0	0.1	0.0	0.1	0.0	0.0	0.0	0.0
To-1(T)	0.0	1.7	0.0	0.8	0.0	0.0	0.0	0.7
Tu-1(C)	0.0	3.6	0.0	1.5	0.0	0.0	0.0	1.5
Tu-2(C)	0.0	4.1	0.0	1.8	0.0	0.0	0.0	1.7
In-1(T)	0.0	2.5	0.0	1.2	0.0	0.0	0.0	1.0
Tu-3(C)	0.0	2.3	0.0	1.1	0.0	0.0	0.0	0.9
Tu-4(C)	0.0	1.7	0.0	1.1	0.0	0.0	0.0	0.6
Tu-5(C)	0.0	1.2	0.0	1.0	0.0	0.0	0.0	0.4
Cl-1(T)	0.0	0.6	0.0	0.6	0.0	0.0	0.0	0.2
El-1(C)	0.0	0.7	0.0	0.7	0.0	0.0	0.0	0.2
El-2(C)	0.0	0.8	0.0	0.9	0.0	0.0	0.0	0.2
El-3(C)	0.0	0.8	0.0	1.0	0.0	0.0	0.0	0.2

Tot: total concentration. HW: Headwater, initial location of the modeling network; T: tributaries; C: control locations

S4. Detail of Monitoring Locations and Input Data

The following is a description of the monitoring locations distributed along the UWER.

Table S3. Sampling location along the UWER.

Locations	Locations names	Coordinates	
		North	East
Ll-1*(HW)	La Laguna River	394759	6677408
Ll-1**(HW)	La Laguna River	394365	6683358
To-1(T)	Toro River	397255	6689022
Tu-1*(C)	Turbio River after the confluence with the La Laguna River	392449	6683762
Tu-1**(C)	Turbio River after the confluence with the La Laguna River	392246	6683826
Tu-2(C)	Turbio River before the confluence with the Incaguaz River	379416	6681792
In-1(T)	Incaguaz River	379474	6681739
Tu-3(C)	Turbio River at Balala	375061	6687928
Tu-4(C)	Turbio River at Huanta	365789	6697409
Tu-5(C)	Turbio River at Varillar	351719	6686505
Cl-1(T)	Claro River	351551	6680808
El-1(C)	Elqui River at Algarrobal	347130	6680835
El-2(C)	Elqui River at Diaguitas	339645	6676589
El-3(C)	Elqui River at Gualliguaica	326211	6677190

*: Campaign 2018; **: Campaign 2019; HW: Headwater, initial location of the modeling network; T: tributaries; C: control locations

S5. Modeled Network Segmentation

Table S4. Designation of segments of the modeled network and their respective distances.

Location	Description	Segment length (m)	Accumulated distance (m)	Distance by section (m)
LL-1*	HW - Segment 1(S1)	2235.7	2235.7	
S2	Segment 2 (S2)	2235.7	4471.3	6825.0
S3	Segment 3 (S3)	2235.7	6707.0	
Ll-1**	HW - Segment 4 (S4)	118.0	6825.0	
To-1	T Segment 5 (S5)	2304.0	9011.0	2340.0
Tu-1	C Segment 6 (S6)	2091.0	4326.7	
S7	Segment 7 (S7)	2091.0	6417.7	
S8	Segment 8 (S8)	2091.0	8508.7	
S9	Segment 9 (S9)	2091.0	10599.7	16728.0
S10	Segment 10 (S10)	2091.0	12690.7	
S11	Segment 11 (S11)	2091.0	14781.7	
S12	Segment 12 (S12)	2091.0	16872.7	
S13	Segment 13 (S13)	2091.0	18963.7	
Tu-2	C Segment 14 (S14)	100.0	19063.7	100.0
In-1	T Segment 15 (S15)	2133.0	21196.7	
S16	Segment 16 (S16)	2133.0	23329.7	8531.0
S17	Segment 17 (S17)	2133.0	25462.7	
S18	Segment 18 (S18)	2132.0	27594.7	
Tu-3	C Segment 19 (S19)	1997.0	29591.7	
S20	Segment 20 (S20)	1997.0	31588.7	
S21	Segment 21 (S21)	1997.0	33585.7	
S22	Segment 22 (S22)	1997.0	35582.7	15974.0
S23	Segment 23 (S23)	1997.0	37579.7	
S24	Segment 24 (S24)	1997.0	39576.7	
S25	Segment 25 (S25)	1996.0	41572.7	
S26	Segment 26 (S26)	1996.0	43568.7	

*: Campaign 2018; **: Campaign 2019; HW Segment: Headwater, starting location of the modeling network; T Segment: tributaries; C segment: control locations for model performance evaluation.

Table S4 continuation. Designation of segments of the modeled network and their respective distances.

Location	Description	Segment length (m)	Accumulated distance (m)	Distance by section (m)
**Tu-4**	C Segment 27 (S27)	2188.0	45756.7	
**S28**	Segment 28 (S28)	2188.0	47944.7	
**S29**	Segment 29 (S29)	2188.0	50132.7	
**S30**	Segment 30 (S30)	2188.0	52320.7	
**S31**	Segment 31 (S31)	2188.0	54508.7	
**S32**	Segment 32 (S32)	2188.0	56696.7	24067.0
**S33**	Segment 33 (S33)	2188.0	58884.7	
**S34**	Segment 34 (S34)	2188.0	61072.7	
**S35**	Segment 35 (S35)	2188.0	63260.7	
**S36**	Segment 36 (S36)	2188.0	65448.7	
**S37**	Segment 37 (S37)	2187.0	67635.7	
**Tu-5**	C. Segment 38 (S38)	1939.0	69574.7	
**S39**	Segment 39 (S39)	1939.0	71513.7	5816.0
**S40**	Segment 40 (S40)	1938.0	73451.7	
**Cl-1**	T Segment 41 (S41)	2423.0	75874.7	2423.0
**El-1**	C Segment 42 (S42)	1950.0	77824.7	
**S43**	Segment 43 (S43)	1951.0	79775.7	
**S44**	Segment 44 (S44)	1951.0	81726.7	9752.0
**S45**	Segment 45 (S45)	1950.0	83676.7	
**El-2**	C Segment 46 (S46)	1950.0	85626.7	
**S47**	Segment 47 (S47)	2073.0	87699.7	
**S48**	Segment 48 (S48)	2073.0	89772.7	
**S49**	Segment 49 (S49)	2073.0	91845.7	
**S50**	Segment 50 (S50)	2073.0	93918.7	14510.0
**S51**	Segment 51 (S51)	2073.0	95991.7	
**S52**	Segment 52 (S52)	2073.0	98064.7	
**El-3**	C Segment 53 (S53)	2072.0	100136.7	
**S-54**	Segment 54 (S54)	100.0	100236.7	100.0

HW: Headwater, starting location of the modeling network; T: tributaries; C Segment: control locations for model performance evaluation.

S6. Input Data for WASP8

The equations for calculating variables and parameters entered by segments during modeling with WASP are presented in Ambrose and Wool [1]. Subsequently, the tables with the input values for calibration and validation are presented.

- Partition Ratio

$$R_p = \frac{C_p(\frac{mg}{L})}{C_d(\frac{mg}{L})} \tag{S1}$$

- Dissolved fraction

$$F_d = \frac{C_d(\frac{mg}{L})}{C_t(\frac{mg}{L})} \tag{S2}$$

Where,

C_p, metal(loid) concentration in particulate phase in water column
C_d, metal(loid) concentration in the dissolved phase in the water column
C_t, metal(loid) total concentration in the water column

- Fischer et al. [2]:

$$D_L\,(m^2/s) = 0.011\frac{U^2w^2}{dU'} \tag{S3}$$

$$U'(m/s) = \sqrt{gdS} \tag{S4}$$

Where,
Q, flow rate (m^3/s);
w, channel width (m);
S, channel slope (m/m).
D, channel depth (m)
U', channel bottom shear velocity (m).
g, acceleration of gravity (m/s^2)
Calculations from the flow rate are estimated with discharge coefficients calculated from observed stream geometry data. The empirical equations are based on the relationship of

streamflow to velocity and depth [1]. The power functions relating to velocity, channel width, and flow depth are presented below.

- $d^* = d = d_{mult} \cdot Q^{d_{exp}}$ (S5)
- $U^* = v = v_{mult} \cdot Q^{v_{exp}}$ (S6)
- $R = d_{mult} \cdot Q^{d_{xp}}$ (S7)
- $B = b_{mult} \cdot Q^{b_{exp}}$ (S8)
- $Q = v \cdot A = v \cdot R \cdot B = (v_{mult} \cdot Q^{v_{exp}}) \cdot (d_{mult} \cdot Q^{v_{exp}}) \cdot \left(b_{mult} \cdot Q^{d_{xp}}\right)$
  $= (v_{mult} \cdot d_{mult} \cdot b_{mult})Q^{v_{exp}+d_{xp}+b_{exp}}$ (S9)

Where it is established that:

$v_{exp} \cdot d_{xp} \cdot b_{exp} = 1$ and $v_{exp} + d_{xp} + b_{exp} = 1$ (S10)

v, velocity (m/s)
R, hydraulic radius or average depth (m)
B, width (m)
v_{mult}. d_{mult} y b_{mult}, empirical coefficients

v_{exp} (=0.4), d_{xp} (=0.6) y b_{xp} (=0.0), empirical exponents for rectangular channel

Below are the tables with the input data for the modeling, including data from the 2018 campaign (calibration) and data from the 2019 campaign (validation).

Table S5. Constituent concentrations, flow rates, and pH.

Locations	Observed water column data												Observed sediments data			
	**Al Tot (mg/L)**	**Al Diss (mg/L)**	**Fe Tot (mg/L)**	**Fe Diss (mg/L)**	**As Tot (µg/L)**	**As Diss (µg/L)**	**Cu Tot (mg/L)**	**Cu Diss (mg/L)**	**SO_4 (mg/L)**	**pH**	**Q obs. m^3/s**	**Q m^3/s sum of affluents**	**Al (%)**	**Fe (%)**	**As (ppm)**	**Cu (ppm)**
Ll-1(HW) (2018)	0.10	<0.002	0.161	0.040	10.63	3.32	0.013	0.006	83.1	8.13	0.92	0.92	2.67	6.99	366.50	361.21
Ll-1(HW) (2019)	0.20	0.05	<0.01	<0.01	12.60	7.35	0.005	0.004	134.1	8.20	1.07	1.07	2.24	8.71	382.50	300.00
To-1(T) (2018)	27.54	18.30	8.980	2.650	305.82	<0.03	11.850	9.620	1099.0	4.50	0.38	0.38	3.05	3.70	75.50	65.00
To-1(T) (2019)	13.69	13.52	8.895	2.570	305.00	14.24	7.111	7.036	958.0	4.43	0.50	0.5	2.04	4.45	101.00	33.50
Tu-1(C) (2018)	6.49	<0.002	2.220	0.070	94.84	0.32	2.550	0.095	360.4	7.58	1.67	1.30	2.97	5.82	335.25	1040.75
Tu-1(C) (2019)	3.03	0.03	2.122	<0.01	84.16	<0.03	1.442	0.036	297.0	7.70	3.22	1.57	2.23	7.05	196.50	994.50
Tu-2(C) (2018)	6.51	0.03	2.300	0.104	105.50	5.21	2.490	0.076	391.6	7.86	3.22	1.30	8.02	5.82	85.50	3736.00
Tu-2(C) (2019)	2.63	0.03	1.056	0.235	52.39	<0.03	1.330	0.026	311.0	8.16	2.10	1.57	2.82	4.62	191.50	2098.50
In-1(T) (2018)	0.68	<0.002	0.347	0.043	3.75	<0.03	0.088	0.037	78.1	7.52	0.72	0.72	3.37	6.24	459.00	4984.25
In-1(T) (2019)	0.32	0.03	0.086	<0.01	<0.03	<0.03	0.025	0.007	124.2	8.15	0.70	0.7	N.d.	N.d.	N.d.	N.d.
Tu-3(C) (2018)	5.06	0.08	1.730	0.047	78.52	4.24	1.850	0.044	283.0	7.99	4.74	2.02	3.36	5.88	389.50	4480.00
Tu-3(C) (2019)	2.29	0.08	1.277	<0.01	54.00	<0.03	1.099	0.029	262.4	8.12	4.78	2.27	2.56	4.92	161.50	1756.00
Tu-4(C) (2018)	3.50	<0.002	1.420	0.048	62.38	3.63	1.120	0.049	275.6	7.95	6.64	2.02	3.24	4.88	352.00	3406.50
Tu-4(C) (2019)	1.94	0.24	0.968	<0.01	49.00	<0.03	0.819	0.024	250.9	8.06	3.00	2.27	2.54	3.95	134.00	1373.50
Tu-5(C) (2018)	3.11	<0.002	1.270	0.054	51.15	3.91	1.000	0.049	251.7	7.91	4.17	2.02	3.17	4.94	334.50	3131.50
Tu-5(C) (2019)	1.60	0.03	0.818	<0.01	37.54	<0.03	0.664	0.019	248.5	7.91	2.81	2.27	2.75	4.29	147.50	1662.50
Cl-1(T) (2018)	0.15	<0.002	0.258	<0.01	4.16	0.64	0.006	0.004	64.1	7.74	2.20	2.2	1.90	3.31	34.50	77.00
Cl-1(T) (2019)	0.03	0.01	<0.01	<0.01	<0.03	<0.03	0.004	0.003	75.7	7.97	1.17	1.17	1.75	3.85	25.00	57.00
El-1(C) (2018)	2.02	0.03	0.843	0.067	29.24	3.12	0.579	0.056	192.5	8.19	4.31	4.22	3.15	5.09	201.50	3093.50
El-1(C) (2019)	1.20	0.05	0.693	<0.01	24.30	<0.03	0.480	0.024	201.6	8.08	4.73	3.44	2.65	4.35	153.00	1425.00
El-2(C) (2018)	2.16	<0.002	1.010	0.109	34.93	6.84	0.660	0.036	193.3	8.18	7.71	4.22	2.77	4.57	189.50	2237.00
El-2(C) (2019)	1.02	0.02	0.484	<0.01	20.60	<0.03	0.427	0.017	193.3	8.30	2.34	3.44	2.81	3.95	131.50	1285.00
El-3(C) (2018)	1.03	<0.002	0.700	0.083	12.70	7.97	0.278	0.031	204.9	7.81	4.66	4.22	3.17	4.87	150.00	2423.00
El-3(C) (2019)	0.42	0.02	0.018	<0.01	1.72	<0.03	0.149	0.020	205.6	7.91	5.40	3.44	3.23	4.18	138.50	1871.00

Q obs.: observed flow rate; N.d.: no data. Tot: Total concentrations; Diss: Dissolved concentration; HW: Headwater, initial location of the modeling network; T: tributaries; C: control locations.

Table S6. Input data for modeling with 2018 campaign data for segment 1 to segment 18.

Locations	Partition Ratio (R_p)				Dissolved Fraction (F_d)				Dispersion coefficient (D_L)				Channel geometry												
	Al	Fe	As	Cu	Al	Fe	As	Cu	S (m/m)	U' (m/s)	D_L (m^2/s)		Q_{imp} (m^3/s)	D (m)	W (m)	A (m^2)	U (m/s)	V_{seg} (m^3)	L (m)	d*	d_{multp}	d_{exp}	V_{exp}	U*	V_{mult}
LL-1*(HW)	49.0	3.0	2.2	1.0	0.02	0.25	0.31	0.49	0.027	0.28	1.23		0.92	0.30	3.30	0.99	0.93	2213	2236	0.30	0.32	0.60	0.40	0.93	0.96
S2	49.0	30.7	295.4	26.0	0.02	0.25	0.31	0.49	0.027	0.28	1.23	D_L-1	0.92	0.30	3.30	0.99	0.93	2213	2236	0.30	0.32	0.60	0.40	0.93	0.96
S3	49.0	30.7	295.4	26.0	0.02	0.25	0.31	0.49	0.027	0.28	1.23		0.92	0.30	3.30	0.99	0.93	2213	2236	0.30	0.32	0.60	0.40	0.93	0.96
LL-1**(HW)	49.0	30.7	295.4	26.0	0.02	0.25	0.31	0.49	0.027	0.28	1.23		0.92	0.30	3.30	0.99	0.93	117	118	0.30	0.32	0.60	0.40	0.93	0.96
To-1(T)	3244.0	30.7	295.4	26.0	0.29	0.02	0.00	0.48	0.045	0.31	5.26	D_L-2	1.30	0.22	6.75	1.51	0.86	3483	2304	0.22	0.19	0.60	0.40	0.86	0.77
Tu-1(C)	3244.0	30.7	295.4	26.0	0.00	0.03	0.00	0.04	0.028	0.25	6.67		1.30	0.22	6.75	1.51	0.86	3161	2091	0.22	0.19	0.60	0.40	0.86	0.77
S7	3244.0	30.7	295.4	26.0	0.00	0.03	0.00	0.04	0.028	0.25	6.67		1.30	0.22	6.75	1.51	0.86	3161	2091	0.22	0.19	0.60	0.40	0.86	0.77
S8	3244.0	30.7	295.4	26.0	0.00	0.03	0.00	0.04	0.028	0.25	6.67		1.30	0.22	6.75	1.51	0.86	3161	2091	0.22	0.19	0.60	0.40	0.86	0.77
S9	3244.0	30.7	295.4	26.0	0.00	0.03	0.00	0.04	0.028	0.25	6.67	D_L-3	1.30	0.22	6.75	1.51	0.86	3161	2091	0.22	0.19	0.60	0.40	0.86	0.77
S10	3244.0	30.7	295.4	26.0	0.00	0.03	0.00	0.04	0.028	0.25	6.67		1.30	0.22	6.75	1.51	0.86	3161	2091	0.22	0.19	0.60	0.40	0.86	0.77
S11	3244.0	30.7	295.4	26.0	0.00	0.03	0.00	0.04	0.028	0.25	6.67		1.30	0.22	6.75	1.51	0.86	3161	2091	0.22	0.19	0.60	0.40	0.86	0.77
S12	3244.0	30.7	295.4	26.0	0.00	0.03	0.00	0.04	0.028	0.25	6.67		1.30	0.22	6.75	1.51	0.86	3161	2091	0.22	0.19	0.60	0.40	0.86	0.77
S13	3244.0	30.7	295.4	26.0	0.00	0.03	0.00	0.04	0.028	0.25	6.67		1.30	0.22	6.75	1.51	0.86	3161	2091	0.22	0.19	0.60	0.40	0.86	0.77
Tu-2(C)	209.0	21.1	19.2	31.7	0.00	0.05	0.05	0.03	0.023	0.22	7.36	D_L-4	1.30	0.22	6.75	1.51	0.86	151	100	0.22	0.19	0.60	0.40	0.86	0.77
In-1(T)	62.3	35.8	17.5	40.7	0.02	0.03	0.05	0.02	0.023	0.26	6.75		2.02	0.30	9.00	2.66	0.76	5669	2133	0.30	0.19	0.60	0.40	0.76	0.57
S16	62.3	35.8	17.5	40.7	0.02	0.03	0.05	0.02	0.023	0.26	6.75	D_L-5	2.02	0.30	9.00	2.66	0.76	5669	2133	0.30	0.19	0.60	0.40	0.76	0.57
S17	62.3	35.8	17.5	40.7	0.02	0.03	0.05	0.02	0.023	0.26	6.75		2.02	0.30	9.00	2.66	0.76	5669	2133	0.30	0.19	0.60	0.40	0.76	0.57
S18	62.3	35.8	17.5	40.7	0.02	0.03	0.05	0.02	0.023	0.26	6.75		2.02	0.30	9.00	2.66	0.76	5667	2132	0.30	0.19	0.60	0.40	0.76	0.57

Ll-1*: sampling location 2018 campaign; Ll-1**: sampling location 2019 campaign; S: channel slope; U′: channel bottom shear velocity; Q: flow rate; d: channel depth; w: channel weigh; A: channel cross-sectional area; U: velocity; V_{seg}: segment volume; L: segment length; d*: empirical channel depth; U:* empirical velocity; d_{multp}: empirical coefficient calculated from d*; d_{exp}: Exponent for depth (rectangular channel cross-section); V_{exp}: Exponent for Velocity (rectangular channel cross-section); V_{mult}: empirical coefficient calculated from U*. HW: Headwater, initial location of the modeling network; T: tributaries; C: control locations.

Table S6 continuation. Input data for modeling with 2018 campaign data for segment 19 to segment 40.

Locations	Partition Ratio (R_p)				Dissolved Fraction (F_d)				Dispersion coefficient (D_L)				Channel geometry												
	Al	Fe	As	Cu	Al	Fe	As	Cu	S (m/m)	U' (m/s)	D_L (m^2/s)		Q (m^3/s)	D (m)	W (m)	A (m^2)	U (m/s)	V_{seg} (m^3)	L (m)	d*	d_{multp}	d_{exp}	V_{exp}	U*	V_{mult}
Tu-3(C)	62.3	35.8	17.5	40.7	0.02	0.03	0.05	0.02	0.020	0.24	7.24		2.02	0.30	9.00	2.66	0.76	5308	1997	0.30	0.19	0.60	0.40	0.76	0.57
S20	62.3	35.8	17.5	40.7	0.02	0.03	0.05	0.02	0.020	0.24	7.24		2.02	0.30	9.00	2.66	0.76	5308	1997	0.30	0.19	0.60	0.40	0.76	0.57
S21	62.3	35.8	17.5	40.7	0.02	0.03	0.05	0.02	0.020	0.24	7.24		2.02	0.30	9.00	2.66	0.76	5308	1997	0.30	0.19	0.60	0.40	0.76	0.57
S22	62.3	35.8	17.5	40.7	0.02	0.03	0.05	0.02	0.020	0.24	7.24	D_L-6	2.02	0.30	9.00	2.66	0.76	5308	1997	0.30	0.19	0.60	0.40	0.76	0.57
S23	62.3	35.8	17.5	40.7	0.02	0.03	0.05	0.02	0.020	0.24	7.24		2.02	0.30	9.00	2.66	0.76	5308	1997	0.30	0.19	0.60	0.40	0.76	0.57
S24	62.3	35.8	17.5	40.7	0.02	0.03	0.05	0.02	0.020	0.24	7.24		2.02	0.30	9.00	2.66	0.76	5308	1997	0.30	0.19	0.60	0.40	0.76	0.57
S25	62.3	35.8	17.5	40.7	0.02	0.03	0.05	0.02	0.020	0.24	7.24		2.02	0.30	9.00	2.66	0.76	5305	1996	0.30	0.19	0.60	0.40	0.76	0.57
S26	62.3	35.8	17.5	40.7	0.02	0.03	0.05	0.02	0.020	0.24	7.24		2.02	0.30	9.00	2.66	0.76	5305	1996	0.30	0.19	0.60	0.40	0.76	0.57
Tu-4(C)	1749.0	28.6	16.2	21.8	0.00	0.03	0.06	0.04	0.019	0.23	7.43		2.02	0.30	9.00	2.66	0.76	5815	2188	0.30	0.19	0.60	0.40	0.76	0.57
S28	1749.0	28.6	16.2	21.8	0.00	0.03	0.06	0.04	0.019	0.23	7.43		2.02	0.30	9.00	2.66	0.76	5815	2188	0.30	0.19	0.60	0.40	0.76	0.57
S29	1749.0	28.6	16.2	21.8	0.00	0.03	0.06	0.04	0.019	0.23	7.43		2.02	0.30	9.00	2.66	0.76	5815	2188	0.30	0.19	0.60	0.40	0.76	0.57
S30	1749.0	28.6	16.2	21.8	0.00	0.03	0.06	0.04	0.019	0.23	7.43		2.02	0.30	9.00	2.66	0.76	5815	2188	0.30	0.19	0.60	0.40	0.76	0.57
S31	1749.0	28.6	16.2	21.8	0.00	0.03	0.06	0.04	0.019	0.23	7.43		2.02	0.30	9.00	2.66	0.76	5815	2188	0.30	0.19	0.60	0.40	0.76	0.57
S32	1749.0	28.6	16.2	21.8	0.00	0.03	0.06	0.04	0.019	0.23	7.43	D_L-7	2.02	0.30	9.00	2.66	0.76	5815	2188	0.30	0.19	0.60	0.40	0.76	0.57
S33	1749.0	28.6	16.2	21.8	0.00	0.03	0.06	0.04	0.019	0.23	7.43		2.02	0.30	9.00	2.66	0.76	5815	2188	0.30	0.19	0.60	0.40	0.76	0.57
S34	1749.0	28.6	16.2	21.8	0.00	0.03	0.06	0.04	0.019	0.23	7.43		2.02	0.30	9.00	2.66	0.76	5815	2188	0.30	0.19	0.60	0.40	0.76	0.57
S35	1749.0	28.6	16.2	21.8	0.00	0.03	0.06	0.04	0.019	0.23	7.43		2.02	0.30	9.00	2.66	0.76	5815	2188	0.30	0.19	0.60	0.40	0.76	0.57
S36	1749.0	28.6	16.2	21.8	0.00	0.03	0.06	0.04	0.019	0.23	7.43		2.02	0.30	9.00	2.66	0.76	5815	2188	0.30	0.19	0.60	0.40	0.76	0.57
S37	1749.0	28.6	16.2	21.8	0.00	0.03	0.06	0.04	0.019	0.23	7.43		2.02	0.30	9.00	2.66	0.76	5813	2187	0.30	0.19	0.60	0.40	0.76	0.57
Tu-5(C)	1554.0	22.5	12.1	19.4	0.00	0.04	0.08	0.05	0.015	0.21	8.36		2.02	0.30	9.00	2.66	0.76	5154	1939	0.30	0.19	0.60	0.40	0.76	0.57
S39	1554.0	22.5	12.1	19.4	0.00	0.04	0.08	0.05	0.015	0.21	8.36	D_L-8	2.02	0.30	9.00	2.66	0.76	5154	1939	0.30	0.19	0.60	0.40	0.76	0.57
S40	1554.0	22.5	12.1	19.4	0.00	0.04	0.08	0.05	0.015	0.21	8.36		2.02	0.30	9.00	2.66	0.76	5151	1938	0.30	0.19	0.60	0.40	0.76	0.57

Ll-1*: sampling location 2018 campaign; Ll-1**: sampling location 2019 campaign; S: channel slope; U′: channel bottom shear velocity; Q: flow rate; d: channel depth; w: channel weigh; A: channel cross-sectional area; U: velocity; V_{seg}: segment volume; L: segment length; d*: empirical channel depth; U:* empirical velocity; d_{multp}: empirical coefficient calculated from d*; d_{exp}: Exponent for depth (rectangular channel cross-section); V_{exp}: Exponent for Velocity (rectangular channel cross-section); V_{mult}: empirical coefficient calculated from U*. HW: Headwater, initial location of the modeling network; T: tributaries; C: control locations.

Table S6 continuation. Input data for modeling with 2018 campaign data for segment 41 to segment 54.

Locations	Partition Ratio (R_p)				Dissolved Fraction (F_d)				Dispersion coefficient (D_L)				Channel geometry												
	Al	Fe	As	Cu	Al	Fe	As	Cu	S (m/m)	U' (m/s)	D_L (m^2/s)		Q (m^3/s)	D (m)	W (m)	A (m^2)	U (m/s)	V_{seg} (m^3)	L (m)	d*	d_{multp}	d_{exp}	V_{exp}	U*	V_{mult}
Cl-1(T)	68.7	11.6	8.4	9.4	0.01	0.08	0.11	0.10	0.015	0.27	5.68	D_L-9	4.22	0.50	10.50	5.28	0.80	12781	2423	0.50	0.21	0.60	0.40	0.80	0.45
El-1(C)	68.7	11.6	8.4	9.4	0.01	0.08	0.11	0.10	0.014	0.26	5.88		4.22	0.50	10.50	5.28	0.80	10286	1950	0.50	0.21	0.60	0.40	0.80	0.45
S43	68.7	11.6	8.4	9.4	0.01	0.08	0.11	0.10	0.014	0.26	5.88		4.22	0.50	10.50	5.28	0.80	10292	1951	0.50	0.21	0.60	0.40	0.80	0.45
S44	68.7	11.6	8.4	9.4	0.01	0.08	0.11	0.10	0.014	0.26	5.88	D_L-10	4.22	0.50	10.50	5.28	0.80	10292	1951	0.50	0.21	0.60	0.40	0.80	0.45
S45	68.7	11.6	8.4	9.4	0.01	0.08	0.11	0.10	0.014	0.26	5.88		4.22	0.50	10.50	5.28	0.80	10292	1950	0.50	0.21	0.60	0.40	0.80	0.45
El-2(C)	1079.0	8.3	4.1	17.6	0.00	0.11	0.20	0.05	0.014	0.26	5.88		4.22	0.50	10.50	5.28	0.80	10286	1950	0.50	0.21	0.60	0.40	0.80	0.45
S47	1079.0	8.3	4.1	17.6	0.00	0.11	0.20	0.05	0.012	0.24	6.35		4.22	0.50	10.50	5.28	0.80	10286	2073	0.50	0.21	0.60	0.40	0.80	0.45
S48	1079.0	8.3	4.1	17.6	0.00	0.11	0.20	0.05	0.012	0.24	6.35		4.22	0.50	10.50	5.28	0.80	10935	2073	0.50	0.21	0.60	0.40	0.80	0.45
S49	1079.0	8.3	4.1	17.6	0.00	0.11	0.20	0.05	0.012	0.24	6.35		4.22	0.50	10.50	5.28	0.80	10935	2073	0.50	0.21	0.60	0.40	0.80	0.45
S50	1079.0	8.3	4.1	17.6	0.00	0.11	0.20	0.05	0.012	0.24	6.35	D_L-11	4.22	0.50	10.50	5.28	0.80	10935	2073	0.50	0.21	0.60	0.40	0.80	0.45
S51	1079.0	8.3	4.1	17.6	0.00	0.11	0.20	0.05	0.012	0.24	6.35		4.22	0.50	10.50	5.28	0.80	10935	2073	0.50	0.21	0.60	0.40	0.80	0.45
S52	1079.0	8.3	4.1	17.6	0.00	0.11	0.20	0.05	0.012	0.24	6.35		4.22	0.50	10.50	5.28	0.80	10935	2073	0.50	0.21	0.60	0.40	0.80	0.45
El-3(C)	514.0	7.4	0.6	8.1	0.00	0.12	0.63	0.11	0.012	0.24	6.35		4.22	0.50	10.50	5.28	0.80	10930	2072	0.50	0.21	0.60	0.40	0.80	0.45
S54	514.0	7.4	0.6	8.1	0.00	0.12	0.63	0.11	0.012	0.24	6.35		4.22	0.50	10.50	5.28	0.80	528	100	0.50	0.21	0.60	0.40	0.80	0.45

Ll-1*: sampling location 2018 campaign; Ll-1**: sampling location 2019 campaign; S: channel slope; U': channel bottom shear velocity; Q: flow rate; d: channel depth; w: channel weigh; A: channel cross-sectional area; U: velocity; V_{seg}: segment volume; L: segment length; d*: empirical channel depth; U:* empirical velocity; d_{multp}: empirical coefficient calculated from d*; d_{exp}: Exponent for depth (rectangular channel cross-section); V_{exp}: Exponent for Velocity (rectangular channel cross-section); V_{mult}: empirical coefficient calculated from U*. HW: Headwater, initial location of the modeling network; T: tributaries; C: control locations.

Table S7. Input data for modeling with 2019 campaign data for segment 1 to segment 18.

Locations	Partition Ratio (R_p)				Dissolved Fraction (F_d)				Dispersion coefficient (D_L)				Channel geometry												
	Al	Fe	As	Cu	Al	Fe	As	Cu	S (m/m)	V' (m/s)	D_L (m^2/s)		Q_{imp} (m^3/s)	D (m)	W (m)	A (m^2)	U (m/s)	V_{seg} (m^3)	L (m)	d*	d_{multp}	d_{exp}	V_{exp}	U*	V_{mult}
LL-1*(HW)	3.4	0.0	0.7	0.2	0.23	1.00	0.58	0.81	0.027	0.35	0.34		1.1	0.5	3.5	1.6	0.7	3680	2236	0.5	0.5	0.6	0.4	0.7	0.6
S2	3.4	0.0	0.7	0.2	0.23	1.00	0.58	0.81	0.027	0.35	0.34	D_L-1	1.1	0.5	3.5	1.6	0.7	3680	2236	0.5	0.5	0.6	0.4	0.7	0.6
S3	3.4	0.0	0.7	0.2	0.23	1.00	0.58	0.81	0.027	0.35	0.34		1.1	0.5	3.5	1.6	0.7	3680	2236	0.5	0.5	0.6	0.4	0.7	0.6
LL-1**(HW)	3.4	0.00	0.7	0.2	0.23	1.00	0.58	0.81	0.027	0.35	0.34		1.1	0.5	3.5	1.6	0.7	194	118	0.5	0.5	0.6	0.4	0.7	0.6
To-1(T)	96.7	211.2	2804.3	39.4	0.01	0.00	0.00	0.02	0.045	0.40	1.53	D_L-2	1.6	0.4	6.6	2.3	0.7	5399	2304	0.4	0.3	0.6	0.4	0.7	0.6
Tu-1(C)	96.7	211.2	2804.3	39.4	0.01	0.00	0.00	0.02	0.028	0.31	1.94		1.6	0.4	6.6	2.3	0.7	4900	2091	0.4	0.3	0.6	0.4	0.7	0.6
S7	96.7	211.2	2804.3	39.4	0.01	0.00	0.00	0.02	0.028	0.31	1.94		1.6	0.4	6.6	2.3	0.7	4900	2091	0.4	0.3	0.6	0.4	0.7	0.6
S8	96.7	211.2	2804.3	39.4	0.01	0.00	0.00	0.02	0.028	0.31	1.94		1.6	0.4	6.6	2.3	0.7	4900	2091	0.4	0.3	0.6	0.4	0.7	0.6
S9	96.7	211.2	2804.3	39.4	0.01	0.00	0.00	0.02	0.028	0.31	1.94		1.6	0.4	6.6	2.3	0.7	4900	2091	0.4	0.3	0.6	0.4	0.7	0.6
S10	96.7	211.2	2804.3	39.4	0.01	0.00	0.00	0.02	0.028	0.31	1.94	D_L-3	1.6	0.4	6.6	2.3	0.7	4900	2091	0.4	0.3	0.6	0.4	0.7	0.6
S11	96.7	211.2	2804.3	39.4	0.01	0.00	0.00	0.02	0.028	0.31	1.94		1.6	0.4	6.6	2.3	0.7	4900	2091	0.4	0.3	0.6	0.4	0.7	0.6
S12	96.7	211.2	2804.3	39.4	0.01	0.00	0.00	0.02	0.028	0.31	1.94		1.6	0.4	6.6	2.3	0.7	4900	2091	0.4	0.3	0.6	0.4	0.7	0.6
S13	96.7	211.2	2804.3	39.4	0.01	0.00	0.00	0.02	0.028	0.31	1.94		1.6	0.4	6.6	2.3	0.7	4900	2091	0.4	0.3	0.6	0.4	0.7	0.6
Tu-2(C)	78.6	3.5	1745.3	50.6	0.01	0.22	0.00	0.02	0.023	0.28	2.14	D_L-4	1.6	0.4	6.6	2.3	0.7	234	100	0.4	0.3	0.6	0.4	0.7	0.6
In-1(T)	27.0	126.7	1799.0	36.9	0.04	0.01	0.00	0.03	0.023	0.31	2.44		2.3	0.4	7.2	3.0	0.8	6456	2133	0.4	0.3	0.6	0.4	0.8	0.5
S16	27.0	126.7	1799.0	36.9	0.04	0.01	0.00	0.03	0.023	0.31	2.44	D_L-5	2.3	0.4	7.2	3.0	0.8	6456	2133	0.4	0.3	0.6	0.4	0.8	0.5
S17	27.0	126.7	1799.0	36.9	0.04	0.01	0.00	0.03	0.023	0.31	2.44		2.3	0.4	7.2	3.0	0.8	6456	2133	0.4	0.3	0.6	0.4	0.8	0.5
S18	27.0	126.7	1799.0	36.9	0.04	0.01	0.00	0.03	0.023	0.31	2.44		2.3	0.4	7.2	3.0	0.8	6453	2132	0.4	0.3	0.6	0.4	0.8	0.5

Ll-1*: sampling location 2018 campaign; Ll-1**: sampling location 2019 campaign; S: channel slope; U′: channel bottom shear velocity; Q: flow rate; d: channel depth; w: channel weigh; A: channel cross-sectional area; U: velocity; V_{seg}: segment volume; L: segment length; d*: empirical channel depth; U:* empirical velocity; d_{multp}: empirical coefficient calculated from d*; d_{exp}: Exponent for depth (rectangular channel cross-section); V_{exp}: Exponent for Velocity (rectangular channel cross-section); V_{mult}: empirical coefficient calculated from U*. HW: Headwater, initial location of the modeling network; T: tributaries; C: control locations.

Table S7 continuation. Input data for modeling with 2019 campaign data for segment 18 to segment 37.

Locations	Partition Ratio (R_p)				Dissolved Fraction (F_d)				Dispersion coefficient (D_L)				Channel geometry												
	Al	Fe	As	Cu	Al	Fe	As	Cu	S (m/m)	V' (m/s)	D_L (m^2/s)		Q (m^3/s)	D (m)	W (m)	A (m^2)	U (m/s)	V_{seg} (m^3)	L (m)	d*	d_{multp}	d_{exp}	V_{exp}	U*	V_{mult}
Tu-3(C)	27.0	126.7	1799.0	36.9	0.04	0.01	0.00	0.03	0.020	0.29	2.62		2.3	0.4	7.2	3.0	0.8	6044	1997	0.4	0.3	0.6	0.4	0.8	0.5
S20	27.0	126.7	1799.0	36.9	0.04	0.01	0.00	0.03	0.020	0.29	2.62		2.3	0.4	7.2	3.0	0.8	6044	1997	0.4	0.3	0.6	0.4	0.8	0.5
S21	27.0	126.7	1799.0	36.9	0.04	0.01	0.00	0.03	0.020	0.29	2.62		2.3	0.4	7.2	3.0	0.8	6044	1997	0.4	0.3	0.6	0.4	0.8	0.5
S22	27.0	126.7	1799.0	36.9	0.04	0.01	0.00	0.03	0.020	0.29	2.62		2.3	0.4	7.2	3.0	0.8	6044	1997	0.4	0.3	0.6	0.4	0.8	0.5
S23	27.0	126.7	1799.0	36.9	0.04	0.01	0.00	0.03	0.020	0.29	2.62	D_L-6	2.3	0.4	7.2	3.0	0.8	6044	1997	0.4	0.3	0.6	0.4	0.8	0.5
S24	27.0	126.7	1799.0	36.9	0.04	0.01	0.00	0.03	0.020	0.29	2.62		2.3	0.4	7.2	3.0	0.8	6044	1997	0.4	0.3	0.6	0.4	0.8	0.5
S25	27.0	126.7	1799.0	36.9	0.04	0.01	0.00	0.03	0.020	0.29	2.62		2.3	0.4	7.2	3.0	0.8	6041	1996	0.4	0.3	0.6	0.4	0.8	0.5
S26	27.0	126.7	1799.0	36.9	0.04	0.01	0.00	0.03	0.020	0.29	2.62		2.3	0.4	7.2	3.0	0.8	6041	1996	0.4	0.3	0.6	0.4	0.8	0.5
Tu-4(C)	7.1	95.8	1632.3	32.7	0.12	0.01	0.00	0.03	0.019	0.28	2.69		2.3	0.4	7.2	3.0	0.8	6622	2188	0.4	0.3	0.6	0.4	0.8	0.5
S28	7.1	95.8	1632.3	32.7	0.12	0.01	0.00	0.03	0.019	0.28	2.69		2.3	0.4	7.2	3.0	0.8	6622	2188	0.4	0.3	0.6	0.4	0.8	0.5
S29	7.1	95.8	1632.3	32.7	0.12	0.01	0.00	0.03	0.019	0.28	2.69		2.3	0.4	7.2	3.0	0.8	6622	2188	0.4	0.3	0.6	0.4	0.8	0.5
S30	7.1	95.8	1632.3	32.7	0.12	0.01	0.00	0.03	0.019	0.28	2.69		2.3	0.4	7.2	3.0	0.8	6622	2188	0.4	0.3	0.6	0.4	0.8	0.5
S31	7.1	95.8	1632.3	32.7	0.12	0.01	0.00	0.03	0.019	0.28	2.69		2.3	0.4	7.2	3.0	0.8	6622	2188	0.4	0.3	0.6	0.4	0.8	0.5
S32	7.1	95.8	1632.3	32.7	0.12	0.01	0.00	0.03	0.019	0.28	2.69	D_L-7	2.3	0.4	7.2	3.0	0.8	6622	2188	0.4	0.3	0.6	0.4	0.8	0.5
S33	7.1	95.8	1632.3	32.7	0.12	0.01	0.00	0.03	0.019	0.28	2.69		2.3	0.4	7.2	3.0	0.8	6622	2188	0.4	0.3	0.6	0.4	0.8	0.5
S34	7.1	95.8	1632.3	32.7	0.12	0.01	0.00	0.03	0.019	0.28	2.69		2.3	0.4	7.2	3.0	0.8	6622	2188	0.4	0.3	0.6	0.4	0.8	0.5
S35	7.1	95.8	1632.3	32.7	0.12	0.01	0.00	0.03	0.019	0.28	2.69		2.3	0.4	7.2	3.0	0.8	6622	2188	0.4	0.3	0.6	0.4	0.8	0.5
S36	7.1	95.8	1632.3	32.7	0.12	0.01	0.00	0.03	0.019	0.28	2.69		2.3	0.4	7.2	3.0	0.8	6622	2188	0.4	0.3	0.6	0.4	0.8	0.5
S37	7.1	95.8	1632.3	32.7	0.12	0.01	0.00	0.03	0.019	0.28	2.69		2.3	0.4	7.2	3.0	0.8	6619	2187	0.4	0.3	0.6	0.4	0.8	0.5

Ll-1*: sampling location 2018 campaign; Ll-1**: sampling location 2019 campaign; S: channel slope; U': channel bottom shear velocity; Q: flow rate; d: channel depth; w: channel weigh; A: channel cross-sectional area; U: velocity; V_{seg}: segment volume; L: segment length; d*: empirical channel depth; U:* empirical velocity; d_{multp}: empirical coefficient calculated from d*; d_{exp}: Exponent for depth (rectangular channel cross-section); V_{exp}: Exponent for Velocity (rectangular channel cross-section); V_{mult}: empirical coefficient calculated from U*. HW: Headwater, initial location of the modeling network; T: tributaries; C: control locations.

Table S7 continuation. Input data for modeling with 2019 campaign data for segment 38 to segment 54.

Locations	Partition Ratio (R_p)				Dissolved Fraction (F_d)				Dispersion coefficient (D_L)				Channel geometry													
	Al	Fe	As	Cu	Al	Fe	As	Cu	S (m/m)	V' (m/s)	D_L (m^2/s)		Q (m^3/s)	D (m)	W (m)	A (m^2)	U (m/s)	V_{seg} (m^3)	L (m)	d*	d_{multp}	d_{exp}	V_{exp}	U*	V_{mult}
Tu-5(C)	46.1	80.8	1250.3	33.6	0.02	0.01	0.00	0.03	0.015	0.25	3.02		2.3	0.4	7.2	3.0	0.8	5869	1939	0.4	0.3	0.6	0.4	0.8	0.5
S39	46.1	80.8	1250.3	33.6	0.02	0.01	0.00	0.03	0.015	0.25	3.02	D_L-8	2.3	0.4	7.2	3.0	0.8	5869	1939	0.4	0.3	0.6	0.4	0.8	0.5
S40	46.1	80.8	1250.3	33.6	0.02	0.01	0.00	0.03	0.015	0.25	3.02		2.3	0.4	7.2	3.0	0.8	5866	1938	0.4	0.3	0.6	0.4	0.8	0.5
Cl-1(T)	24.0	68.3	809.0	19.4	0.04	0.01	0.00	0.05	0.015	0.32	1.27	D_L-9	3.4	0.7	8.4	5.7	0.6	13892	2423	0.7	0.3	0.6	0.4	0.6	0.4
El-1(C)	24.0	68.3	809.0	19.4	0.04	0.01	0.00	0.05	0.014	0.31	1.31		3.4	0.7	8.4	5.7	0.6	11180	1950	0.7	0.3	0.6	0.4	0.6	0.4
S43	24.0	68.3	809.0	19.4	0.04	0.01	0.00	0.05	0.014	0.31	1.31		3.4	0.7	8.4	5.7	0.6	11186	1951	0.7	0.3	0.6	0.4	0.6	0.4
S44	24.0	68.3	809.0	19.4	0.04	0.01	0.00	0.05	0.014	0.31	1.31	D_L-10	3.4	0.7	8.4	5.7	0.6	11186	1951	0.7	0.3	0.6	0.4	0.6	0.4
S45	24.0	68.3	809.0	19.4	0.04	0.01	0.00	0.05	0.014	0.31	1.31		3.4	0.7	8.4	5.7	0.6	11186	1950	0.7	0.3	0.6	0.4	0.6	0.4
El-2(C)	59.1	47.4	685.7	24.9	0.02	0.02	0.00	0.04	0.014	0.31	1.31		3.4	0.7	8.4	5.7	0.6	11180	1950	0.7	0.3	0.6	0.4	0.6	0.4
S47	59.1	47.4	685.7	24.9	0.02	0.02	0.00	0.04	0.012	0.28	1.41		3.4	0.7	8.4	5.7	0.6	11180	2073	0.7	0.3	0.6	0.4	0.6	0.4
S48	59.1	47.4	685.7	24.9	0.02	0.02	0.00	0.04	0.012	0.28	1.41		3.4	0.7	8.4	5.7	0.6	11885	2073	0.7	0.3	0.6	0.4	0.6	0.4
S49	59.1	47.4	685.7	24.9	0.02	0.02	0.00	0.04	0.012	0.28	1.41		3.4	0.7	8.4	5.7	0.6	11885	2073	0.7	0.3	0.6	0.4	0.6	0.4
S50	59.1	47.4	685.7	24.9	0.02	0.02	0.00	0.04	0.012	0.28	1.41	D_L-11	3.4	0.7	8.4	5.7	0.6	11885	2073	0.7	0.3	0.6	0.4	0.6	0.4
S51	59.1	47.4	685.7	24.9	0.02	0.02	0.00	0.04	0.012	0.28	1.41		3.4	0.7	8.4	5.7	0.6	11885	2073	0.7	0.3	0.6	0.4	0.6	0.4
S52	59.1	47.4	685.7	24.9	0.02	0.02	0.00	0.04	0.012	0.28	1.41		3.4	0.7	8.4	5.7	0.6	11885	2073	0.7	0.3	0.6	0.4	0.6	0.4
El-3(C)	18.0	0.8	56.3	6.6	0.05	0.56	0.02	0.13	0.012	0.28	1.41		3.4	0.7	8.4	5.7	0.6	11879	2072	0.7	0.3	0.6	0.4	0.6	0.4
S54	18.0	0.8	56.3	6.6	0.05	0.56	0.02	0.13	0.012	0.28	1.41		3.4	0.7	8.4	5.7	0.6	573	100	0.7	0.3	0.6	0.4	0.6	0.4

Ll-1*: sampling location 2018 campaign; Ll-1**: sampling location 2019 campaign; S: channel slope; U′: channel bottom shear velocity; Q: flow rate; D: channel depth; W: channel weigh; A: channel cross-sectional area; U: velocity; V_{seg}: segment volume; L: segment length; d*: empirical channel depth; U:* empirical velocity; d_{multp}: empirical coefficient calculated from d*; d_{exp}: Exponent for depth (rectangular channel cross-section); V_{exp}: Exponent for Velocity (rectangular channel cross-section); V_{mult}: empirical coefficient calculated from U*. HW: Headwater, initial location of the modeling network; T: tributaries; C: control locations.

S7. Literature review for partition coefficient (K_d) values.

The literature review to identify the ranges of variation of the partition coefficient (K_d) is presented below in Table S8.

Table S8. Literature review for partition coefficient (K_d) values.

Authors	Metal(loid)s				Water body type	pH
	Al	Fe	As	Cu		
Caruso [3]	-	-	1-5*10^5	3*10^5	Upper Tenmile Creek, EEUU	5-8.9
van Vliet et al. [4]	-	-	10^4	5*10^4	Meuse River, Europa	7.5-7.9
Garneau et al. [5]	-	-	$10^{4.15}$-$10^{4.28}$	-	Garonne River, France	-
Velleux et al. [6]	-	-	-	$10^{3.24}$	Arkansas River basin (California Gulch, Colorado, USA)	6
Allison y Allison [7]	-	-	10^2 -10^4 *-10^6	$10^{3.1}$ -$10^{4.7}$ *-$10^{6.1}$	Streams, rivers, and lakes, USA	4-9
Jung et al. [8]**	10^3	-	-	$10^{2.9}$	Chonam-ri - Sagok-ri creeks, South Korea	2.9-7
Sedeño-Díaz et al. [9]**	-	$10^{4.2}$ -$10^{4.6}$ *-$10^{4.4}$	$10^{1.9}$ -$10^{2.2}$ *-$10^{2.45}$	$10^{3.2}$ -$10^{3.4}$*-$10^{3.96}$	Texcoco Lake, Mexico	Alcaline
Hamad et al. [10]	6.2*10^5 - 13.3*10^6	3*10^5 - 15.2*10^6	2*10^3 -1.2*10^6	3.3*10^5 -2.1*10^6	Diyala – Tigris Rivers, Bagdad, Iraq	7.4-8.3
Duc et al. [11]	-	$10^{5.8}$	$10^{4.9}$	$10^{5.4}$	Day River, Vietnam	6.9-8.4
Zeng et al. [12]	-	-	-	$10^{4.1}$-$10^{4.7}$-$10^{5.6}$	Mun River, Thailand	6.1-8.5
Huo y Chen in Zeng et al. [12,13]	-	-	-	$10^{4.1}$-$10^{4.2}$	Yangtze-Jialingjiang Rivers, China	-
Zeng et al. [13]	-	-	-	$10^{2.9}$-$10^{4.6}$-$10^{5.3}$	Zhuijian g River, China	6.4-8.8
Drndarski et al., 1990 in Zeng et al. [13]	-	-	-	$10^{3.9}$	Sava River, India	-
Li et al., 2018 in Zeng et al. [13]	-	-	-	$10^{4.7}$	Beijing River, China	-
Liu et al., 2018 in Zeng [13]	-	-	-	$10^{4.9}$	Río Zhujiang alto, China	-
Feng et al. [14]**	-		4.7*10^3	1.7*10^4	Yangtze Estuary, China	-

*: Median values in Allison and Allison [7]; **: Approximate mean values obtained from Figure. 5 in Jung et al. [8]; Figure 2 in Feng et al. [14]; and Figure 5 in Sedeño-Díaz et al. [9].

S8. Parameters and Constants

All parameters and constants used in the model, along with their variation ranges and the selected or calibrated values, are detailed in the Table S9 and Table S10.

Table S9. Parameters considered for the model, ranges of variation, and selected/calibrated values

	Group	Description	Range	Source	Selected/calibrated value
Defined parameter	Chemical	Partition ratio (R_p).	-	Equation 2 (main text)	Calculated from observed data for each segment.
	Solids	Shear stress exponent for cohesive resuspension.	2	Literature values [15,16] for TSS, silt type [17].	2
		Critical shear stress for cohesive bed erosion.	1-2 N/m^2		2 N/m^2
		Shear stress multiplier for cohesive resuspension.	1 g/m^2/sec		1 g/m^2/sec
	Hydraulic	Dispersion coefficient.	According to the study area	Fischer et al. [2] is the representative equation [18].	Calculated from observed data for each segment
To calibrate	Solids	TSS sedimentation velocity (V_s).	0 - 50 m/d	Literature value [15,16,19,20] for TSS, silt type [17].	0.5 m/d
		TSS resuspension velocity (V_r).	10^{-4} - 10^{-3} m/d	Literature value [15,16,19,20]	10^{-4} m/d

TSS: Total suspended solids.

Table S10. Description of the constants entered in the model, with their respective ranges of variation and selected or calibration values.

	Group	Description	Range	Source	Selected/calibrated value
Defined constants	State variables	Particle density	1.27 – 4.0 g/cm^3	WASP Manual [16] and Knightes et al. [15]: 1.27 g/cm^3 (organic Matter); 2.65 g/cm^3 (siliceous Minerals); 4.0 g/cm^3 (garnet Sand).	2.65 g/cm^3
	Kinetic time step	Advection Calculation option	0	Option 0, default, finite difference calculation “backward” mode.	0
		Maximum oscillation of the mass balance check	0.01	WASP Manual [16]	0.01
	General	Type of water body	1	WASP Manual [16]	1, surface water
	Transport of solids	Solid calculation option (TSS)	0 - 1	WASP Manual [16]	0, allows manual entry of V_s and V_r.
		Particle diameter for solid (TSS)	3.9 – 62.5 μm	WASP Manual [16]. Representative diameter for silt [17].	12.6 μm (median value)
		Critical fraction of cohesive sediment (TSS).	0 - 1	Literature value [15,16], silt type [17].	0.2
		Critical shear stress for cohesive bed erosion (TSS)	0.5 – 8 N/m^2		2 N/m^2
		Shear stress multiplier for cohesive resuspension (TSS)	0.01 – 100 $g/m^2/sec$		1 $g/m^2/sec$
		Shear stress exponent for cohesive resuspension (TSS)	1.6 – 4		2
		Critical shear stress multiplier for non-cohesive resuspension (TSS)	0.5 – 1.5		1
		Shear stress exponent for non-cohesive resuspension (TSS)	1.5 – 2		1.5
		Shear stress multiplier for non-cohesive resuspension (TSS)	0.5 – 1.5		1
		Lower critical shear stress for solid (TSS)	0 – 0.05 N/m^2		0 N/m^2
		Upper critical shear stress for solid (TSS)	0.01 – 0.2 N/m^2		0.2 N/m^2
		Shear stress exponent for solid deposition (TSS)	0 – 2		1
To calibrate	Chemical partitioning	Partition coefficient, Al (at TSS)	10^2 – 10^6 L/kg	Literature value. Range of K_d values used for rivers and streams[1]	$10^{5.0}$ L/kg
		Partition coefficient, Fe (at TSS)			$10^{5.6}$ L/kg
		Partition coefficient, As (at TSS)			$10^{4.8}$ L/kg
		Partition coefficient, Cu (at TSS)			$10^{5.0}$ L/kg

TSS: Total suspended solids. [1]: Bibliographic review (see Supplementary Material).

S9. Model Performance Assessment

The performance indicators were as follows:

- Relative root mean square error (RRMSE)

$$\mathrm{RRMSE} = \frac{\mathrm{RMSE}}{\mathrm{O_{avg}}} * 100 \qquad \text{(S11)}$$

being RMSE the root mean square error, obtained as

$$\mathrm{RMSE} = \sqrt{\frac{\sum_{i=1}^{n}(P_i - O_i)^2}{n}} \qquad \text{(S12)}$$

where O_{avg} (mg/L) is the mean of observed data, Pi (mg/L) is the modeled value, Oi (mg/L) is the observed value, and n is the total number of data pairs (modeled and observed). While RMSE has the units of the variable of interest, RRMSE is expressed as a percentage and allows for comparison between data of different scales. The lower the value (better fit), the lower the residual error variation between the observed and modeled data.

- Coefficient of determination (R^2)

$$R^2 = \left[\frac{\sum_{i=1}^{n}(O_i - O_{avg})(P_i - P_{avg})}{\sqrt{\sum_{i=1}^{n}(O_i - O_{avg})^2 \sum_{i=1}^{n}(P_i - P_{avg})^2}}\right]^2 \qquad \text{(S13)}$$

where P_{avg} is the mean of the modeled data.
The coefficient represents the fit of a simple linear regression model between the observed and modeled data. The range for R^2 varies between 0 and 1, where the higher the value, the better the linear correlation between the data and, therefore, the lower the model error. An acceptable range is considered to be over 0.5 [21].

- Index of agreement (d)

Willmot [22] proposed the index as a standardized measure of the model error, with values varying between 1 (perfect correlation) and 0 (zero correlation).

$$d = 1 - \frac{\sum_{i=1}^{n}(O_i - P_i)^2}{\sum_{i=1}^{n}\left(|P_i - O_{avg}| + |O_i - O_{avg}|\right)^2} \qquad \text{(S14)}$$

References

(1) Ambrose, R. B.; Wool, T. WASP8 Stream Transport - Model Theory and User's Guide - Supplement to Water Quality Analysis Simulation Program (WASP) User Documentation., 2017. https://www.epa.gov/sites/default/files/2018-05/documents/stream-transport-user-guide.pdf (accessed 2023-07-01).

(2) Fischer, H.; List, E.; Koh, R.; Imberger, J.; Brooks, N. *Mixing in Inland and Coastal Waters*; Academic Press Inc, 1979.

(3) Caruso, B. Modeling Metals Transport and Sediment/Water Interactions in a Mining Impacted Mountain Stream. *JAWRA- Journal of the American Water Resources Association* **2004**, *40* (6), 1603–1615.

(4) van Vliet, M. T. H.; Zwolsman, J. J. G. Impact of Summer Droughts on the Water Quality of the Meuse River. *Journal of Hydrology* **2008**, *353* (1), 1–17. https://doi.org/10.1016/j.jhydrol.2008.01.001.

(5) Garneau, C.; Sauvage, S.; Probst, A.; Sánchez-Pérez, J. M. Modelling of Trace Metal Transfer in a Large River under Different Hydrological Conditions (the Garonne River in Southwest France). *Ecological Modelling* **2015**, *306*, 195–204. https://doi.org/10.1016/j.ecolmodel.2014.09.011.

(6) Velleux, M. L.; Julien, P. Y.; Rojas-Sanchez, R.; Clements, W. H.; England, J. F. Simulation of Metals Transport and Toxicity at a Mine-Impacted Watershed: California Gulch, Colorado. *Environ. Sci. Technol.* **2006**, *40* (22), 6996–7004. https://doi.org/10.1021/es0608592.

(7) Allison, J. D.; Allison, T. L. *Partition coefficients for metals in surface water, soil, and waste*. ResearchGate. https://www.researchgate.net/publication/237633308_Partition_coefficients_for_metals_in_surface_water_soil_and_waste (accessed 2024-12-04).

(8) Jung, H.-B.; Yun, S.-T.; Mayer, B.; Kim, S.-O.; Park, S.-S.; Lee, P.-K. Transport and Sediment–Water Partitioning of Trace Metals in Acid Mine Drainage: An Example from the Abandoned Kwangyang Au–Ag Mine Area, South Korea. *Environ Geol* **2005**, *48* (4), 437–449. https://doi.org/10.1007/s00254-005-1257-7.

(9) Sedeño-Díaz, J. E.; López-López, E.; Mendoza-Martínez, E.; Rodríguez-Romero, A. J.; Morales-García, S. S. Distribution Coefficient and Metal Pollution Index in Water and Sediments: Proposal of a New Index for Ecological Risk Assessment of Metals. *Water* **2020**, *12* (1), 29. https://doi.org/10.3390/w12010029.

(10) Hamad, S. H.; Schauer, J. J.; Shafer, M. M.; Al-Raheem, E. A.; Satar, H. The Distribution between the Dissolved and the Particulate Forms of 49 Metals across the Tigris River, Baghdad, Iraq. *The Scientific World Journal* **2012**, *2012*, 246059. https://doi.org/10.1100/2012/246059.

(11) Duc, T. A.; Loi, V. D.; Thao, T. T. Partition of Heavy Metals in a Tropical River System Impacted by Municipal Waste. *Environ Monit Assess* **2013**, *185* (2), 1907–1925. https://doi.org/10.1007/s10661-012-2676-z.

(12) Zeng, J.; Han, G.; Yang, K. Assessment and Sources of Heavy Metals in Suspended Particulate Matter in a Tropical Catchment, Northeast Thailand. *Journal of Cleaner Production* **2020**, *265*, 121898. https://doi.org/10.1016/j.jclepro.2020.121898.

(13) Zeng, J.; Han, G.; Wu, Q.; Tang, Y. Heavy Metals in Suspended Particulate Matter of the Zhujiang River, Southwest China: Contents, Sources, and Health Risks. *International Journal of Environmental Research and Public Health* **2019**, *16* (10), 1843. https://doi.org/10.3390/ijerph16101843.

(14) Feng, C.; Guo, X.; Yin, S.; Tian, C.; Li, Y.; Shen, Z. Heavy Metal Partitioning of Suspended Particulate Matter–Water and Sediment–Water in the Yangtze Estuary. *Chemosphere* **2017**, *185*, 717–725. https://doi.org/10.1016/j.chemosphere.2017.07.075.

(15) Knightes, C. D.; Ambrose, R. B.; Avant, B.; Han, Y.; Acrey, B.; Bouchard, D. C.; Zepp, R.; Wool, T. Modeling Framework for Simulating Concentrations of Solute Chemicals, Nanoparticles, and Solids in Surface Waters and Sediments: WASP8 Advanced Toxicant Module. *Environmental Modelling & Software* **2019**, *111*, 444–458. https://doi.org/10.1016/j.envsoft.2018.10.012.

(16) Ambrose, B.; Avant, B.; Han, Y.; Knightes, C.; Wool, T. Water Quality Assessment Simulation Program (WASP8): Upgrades to the Advanced Toxicant Module for Simulating Dissolved Chemicals, Nanomaterials, and Solids, 2017. https://cfpub.epa.gov/si/si_public_record_report.cfm?dirEntryId=338180&Lab=NERL (accessed 2023-01-07).

(17) Díaz, J. A.; Castillo, D.; Oyarzún, R.; Briso, A.; Montecinos, M.; Pastén, P. The Dynamics of Arsenic and Copper in Solid and Aqueous Phases in Reactive Confluences Receiving Acid Drainage: The Role of Turbidity and Particle Size. *Environmental Pollution* **2023**, *327*, 121449. https://doi.org/10.1016/j.envpol.2023.121449.

(18) Salerno, F.; Rogora, M.; Balestrini, R.; Lami, A.; Tartari, G. A.; Thakuri, S.; Godone, D.; Freppaz, M.; Tartari, G. Glacier Melting Increases the Solute Concentrations of Himalayan Glacial Lakes. *Environ. Sci. Technol.* **2016**, *50* (17), 9150–9160. https://doi.org/10.1021/acs.est.6b02735.

(19) Chapra, S. C. *Surface Water-Quality Modeling*; Waveland Press: United States of America, 2008.

(20) Bitar Alvarez, J. D.; Camacho, L. A. Implementación de un modelo de transporte de metales pesados en el Río Magdalena tramo Girardot - Honda. Memoria para optar al Título de Magíster en Ingeniería Civil. Universidad de Los Andes, Bogotá, Colombia, 2005. https://repositorio.uniandes.edu.co/entities/publication/4be1c1dc-f4ed-40f9-8d21-1a49848dd99f (accessed on 7 Sep-tember 2023).

(21) Chueh, Y.-Y.; Fan, C.; Huang, Y.-Z. Copper Concentration Simulation in a River by SWAT-WASP Integration and Its Application to Assessing the Impacts of Climate Change and Various Remediation Strategies. *Journal of Environmental Management* **2021**, *279*, 111613. https://doi.org/10.1016/j.jenvman.2020.111613.

(22) Willmott, C. J. On the Validation of Models. *Physical Geography* **1981**, *2* (2), 184–194. https://doi.org/10.1080/02723646.1981.10642213.
